# Review of the Interfacial Structure and Properties of Surfactants in Petroleum Production and Geological Storage Systems from a Molecular Scale Perspective

**DOI:** 10.3390/molecules29133230

**Published:** 2024-07-08

**Authors:** Jihui Jia, Shu Yang, Jingwei Li, Yunfeng Liang, Rongjuan Li, Takeshi Tsuji, Ben Niu, Bo Peng

**Affiliations:** 1 State Key Laboratory of Shale Oil and Gas Enrichment Mechanisms and Effective Development, Beijing 100083, China; 2 Unconventional Petroleum Research Institute, China University of Petroleum (Beijing), Beijing 102249, China; 3 International Institute for Carbon-Neutral Energy Research (I^2^CNER), Kyushu University, Fukuoka 8190395, Japan; 4 Department of Systems Innovation, Graduate School of Engineering, The University of Tokyo, Tokyo 1138656, Japan; 5 School of Urban Construction, Zhejiang Shuren University, Hangzhou 310015, China; 6 CNPC Engineering Technology Research Company Limited, Tianjin 300451, China

**Keywords:** enhanced oil recovery, CO_2_ foam, microemulsion, surfactant monolayers, interfacial properties, molecular dynamics simulation

## Abstract

Surfactants play a crucial role in tertiary oil recovery by reducing the interfacial tension between immiscible phases, altering surface wettability, and improving foam film stability. Oil reservoirs have high temperatures and high pressures, making it difficult and hazardous to conduct lab experiments. In this context, molecular dynamics (MD) simulation is a valuable tool for complementing experiments. It can effectively study the microscopic behaviors (such as diffusion, adsorption, and aggregation) of the surfactant molecules in the pore fluids and predict the thermodynamics and kinetics of these systems with a high degree of accuracy. MD simulation also overcomes the limitations of traditional experiments, which often lack the necessary temporal–spatial resolution. Comparing simulated results with experimental data can provide a comprehensive explanation from a microscopic standpoint. This article reviews the state-of-the-art MD simulations of surfactant adsorption and resulting interfacial properties at gas/oil–water interfaces. Initially, the article discusses interfacial properties and methods for evaluating surfactant-formed monolayers, considering variations in interfacial concentration, molecular structure of the surfactants, and synergistic effect of surfactant mixtures. Then, it covers methods for characterizing microstructure at various interfaces and the evolution process of the monolayers’ packing state as a function of interfacial concentration and the surfactants’ molecular structure. Next, it examines the interactions between surfactants and the aqueous phase, focusing on headgroup solvation and counterion condensation. Finally, it analyzes the influence of hydrophobic phase molecular composition on interactions between surfactants and the hydrophobic phase. This review deepened our understanding of the micro-level mechanisms of oil displacement by surfactants and is beneficial for screening and designing surfactants for oil field applications.

## 1. Introduction

The utilization of enhanced oil recovery (EOR) technology often follows the main production phase, also known as water flooding, to achieve a substantial augmentation in oil extraction [1]. It is an effective way to extract oil and gas from unconventional reservoirs as well [2]. The process entails the introduction of chemicals into geological reservoirs that are not naturally occurring, including carbon dioxide (CO_2_) [3], steam [4], and chemical agents such as surfactants and polymers [5,6,7]. Therefore, it is imperative to enhance our understanding of the dynamics of multicomponent complexes, such as microemulsions [8] and foams [9], inside porous media that inherently possess numerous interfaces. Under this circumstance, two major scenarios are perceived. These scenarios involve the water–oil interface, which refers to the contact between two immiscible liquids in microemulsion, and the gas–water interface, which refers to the interface between a gas phase and a liquid phase in foam systems. Moreover, the displacement efficiency in the EOR process is dependent on surface wettability (i.e., contact angles), pore morphology, and remaining oil saturation [7]. 

**Figure 1:**
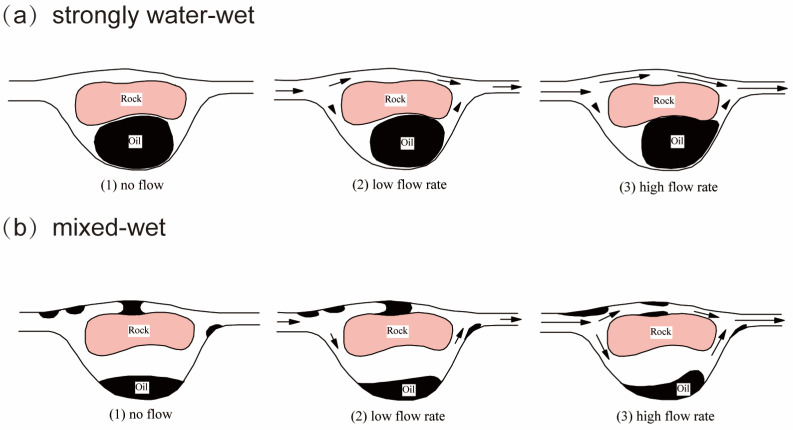
A schematic diagram of possible mechanisms of entrapment and fluid distribution in a porous medium with different flow rates. The black arrows indicate the flow directions. (**a**) strongly water-wet conditions, wherein a water flow path in narrow pores is formed, and the oil in large pores (but small throats) is bypassed; (**b**) mixed wet conditions, showing a different displacement mechanism where the oil is attached to the oil-wetting area [10].

**Figure 2:**
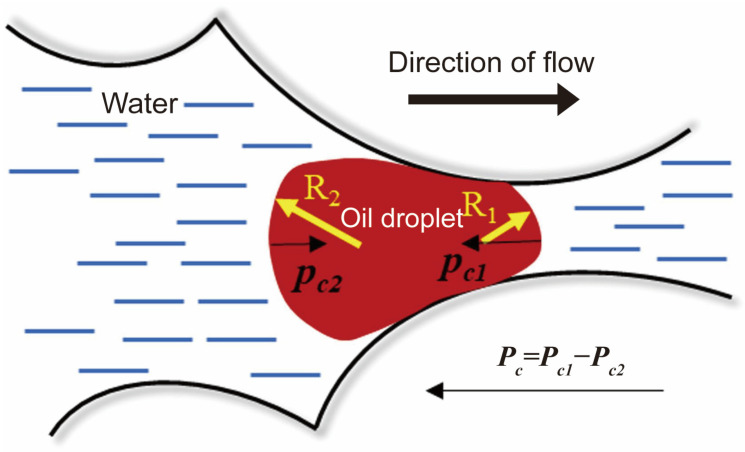
A schematic diagram of the Jamin effect at the pore throat (adapted from [11]). The oil droplet is squeezed in the pore throat and retained by capillary forces.

Figure 1 illustrates possible processes of entrapment and distribution of residual oil in a porous medium [10]. The oil phase possesses a comparatively high viscosity compared to the formation water present in the reservoirs. In strongly water-wet conditions, residual oil (i.e., non-wetting phase) is trapped in large pores by a bypassing mechanism, i.e., the water flow path in narrow pores can be formed, and the oil in large pores (but small throats) can be bypassed. The oil droplet is trapped at the pore throat by capillary force due to the Jamin effect, which is defined as the resistance to liquid flow through capillaries due to the presence of droplets [11], as shown in Figure 2. In contrast, the residual oil exhibits a non-continuous distribution within the porous medium in mixed wet conditions. Permeability to the water phase might increase if oil is redistributed. Bridges of oil that impede permeability at low flow rates can be fractured at increased flow rates. The capillary force is inversely proportional to the capillary number (*Ca*), and the latter can be defined as [5]:

(1) $$Ca=\frac{\mu v}{\gamma \cos\theta }$$

where μ and v represent the viscosity and the flow rate of the displacing phase. γ is the interfacial tension (IFT) between the aqueous phase and the oleic phase. θ denotes the contact angle of oil–water–mineral systems. A lower oil–water IFT helps to relieve the Jamin effect and enhances oil recovery in unconventional reservoirs. To facilitate the advancement of EOR techniques, it is necessary to gain a comprehensive understanding of the efficient reduction of IFT at the oil–water interface, effective control of the surface-active agent (surfactant) adsorption, and accurate assessment of the wetting properties of reservoir rocks [12]. It should be noted that the present review does not delve into the latter aspect, and readers interested in this matter are advised to refer to the work by Ahmadi and coworkers [13]. An alternative strategy involves enhancing sweep efficiency to mitigate the occurrence of fingering. The objective can be accomplished by either enhancing the mobility of residual oil by heating (e.g., injecting steam), which reduces the viscosity of the oil phase [14] or by limiting the mobility of the injectant by raising the viscosity of the displacing phase, such as foam flooding [7].

**Figure 3:**
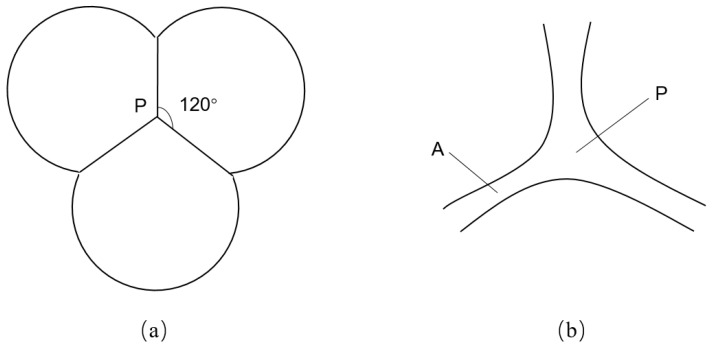
A two-dimensional schematic diagram of foam film’s plateau junction. (**a**) plateau junction of three bubbles; (**b**) enlarged view of plateau junction. “P” denotes a point at the plateau junction (i.e., the plateau node), whereas “A” denotes a point within the foam film (i.e., the plateau border) [16].

**Figure 4:**
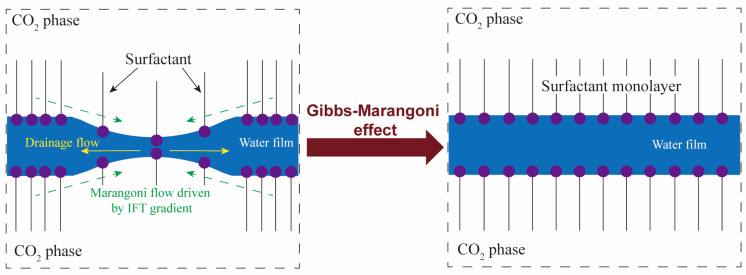
A diagrammatic drawing for the Gibbs-Marangoni effect. The left side depicts that when the liquid film is deformed under external disturbance, a drainage flow and an IFT gradient are generated. The right side illustrates that the liquid film is recovered due to the Gibbs-Marangoni effect, and the surfactant molecules are evenly distributed again at the surface. The purple solid spheres and black sticks represent the headgroups and carbon tails of surfactants, respectively [19].

Foam stability is a significant challenge in the application of foam flooding, and it is subject to influence from multiple factors. Two interfacial phenomena, namely Laplace capillary suction (which controls static stability) and the Gibbs-Marangoni effect (which controls dynamic stability), are particularly important in controlling foam stability [15]. Figure 3 illustrates a microscopic mechanism for the static stability of CO_2_ foam. Point P represents the junction of three bubbles near each other, called the plateau junction (i.e., the plateau node). Because the IFT between gas and water phases causes a pressure difference to exist across a curved surface, the pressure is greater on the concave side (i.e., on the inside of a bubble). The liquid pressure at the curved surface (point P) differs from that at point A within the foam film (i.e., the plateau border). It is subjected to excess pressure ∆p, which can be defined as [16]:

(2) $$∆p=\frac{2\gamma }{R}$$

where R is the radius of the curvature and γ is the IFT at the gas–water interface. This is the Young-Laplace equation. As is observed, the radius of curvature is relatively small at the plateau junction (point P), while the radius of curvature is relatively large at the plateau border (point A), indicating that the pressure at point P in the foam film is smaller than that at point A. Thus, the liquid will automatically flow from points A to P, gradually thinning the foam film. This is one of the liquid discharge processes of the foam film, termed Laplace capillary suction [17]. A lower IFT causes less liquid to drain at the gas–water interface, thus facilitating the generation of foam and the preservation of a larger interfacial area, which are essential for keeping the foam stable. In addition, CO_2_ molecules can penetrate the CO_2_–water interface and diffuse from small bubbles into big bubbles. This process leads to Ostwald ripening, which is detrimental to the foam stability. A surfactant-stabilized CO_2_–water interface can inhibit this phenomenon. Foam texture also plays a vital role in foam stability [17]. Since the interfacial elasticity of the surfactant monolayers and the shear viscosity of the foam film counteract the effect of a mechanical perturbation, a high interfacial elasticity, and a large shear viscosity can be very helpful for the dynamic stability of CO_2_ foam [18,19]. The interfacial elasticity can be expressed by the following equation [20,21]:

(3) $$E=\frac{d\gamma }{dlnA}$$

where γ denotes the IFT at the interface and *A* represents the geometric area of the interface. As demonstrated in Figure 4, when a surfactant-stabilized film experiences a sudden disturbance, the existence of an IFT gradient causes the surfactant molecules to spread from regions with low IFT to regions with high IFT. This behavior compels water molecules to move in a direction opposite to the flow of liquid drainage, which is termed the Gibbs-Marangoni effect. With a large interfacial elasticity, the interfaces can rapidly restore their original flatness after being disturbed by applied forces. This indicates that the interface has high dynamic stability. 

Surfactants are one of the most commonly used chemical agents in EOR methods [22,23]. They have one part that has an affinity for nonpolar media (hydrophobic carbon tails) and one that has an attraction for polar media (hydrophilic or ionic headgroups). This amphiphilic property allows them to adsorb onto the interfaces that are defined as a transition area in two-phase dispersions like foam, microemulsions, and suspensions [24]. Meanwhile, they can also increase the pore fluids’ viscosity [25]. Based on the nature of the polar headgroups, surfactants can be classified into different categories: anionic (negatively charged), cationic (positively charged), nonionic (electrically neutral), and zwitterionic (carry both a positive and a negative charge) surfactants. Depending on the attachment position of the hydrophilic headgroups on the alkyl chains, they can also be categorized into single-tail and multiple-tail structure surfactants [26]. Surfactant types and structures have been varied, and the correlations between their structure and interfacial properties have been developed [19,26]. The molecules in the intermediate region undergo imbalance pulls by bulk phases, leading to the occurrence of IFT. Adsorption of surfactants at the interfaces can significantly reduce the IFT values [27], and the physicochemical properties in this region are extremely important in all kinds of petroleum recovery and processing operations [28,29]. We are still facing many problems, like how to achieve good miscibility in a microemulsion (i.e., oil–water interface) system [30] and mitigate the surfactant loss due to the adsorption on the mineral surface [31] under reservoir conditions, and the stability issue of a foam (i.e., gas–water interface) system remains to be solved for engineering applications [32]. Understanding the dynamics of surfactant adsorption and the influence of surfactant chemical structure on adsorption behaviors at various interfaces has been a significant thrust in the research area. 

**Figure 5:**
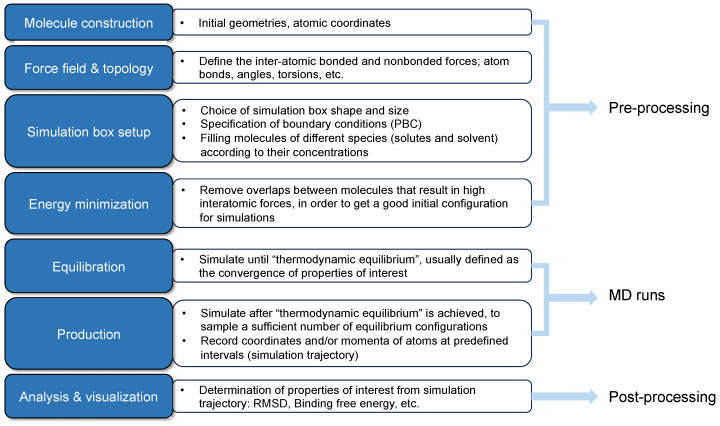
General workflow of molecular dynamics simulations (adapted from [37]).

The investigations on the interfacial performance of surfactant-formed monolayers at the gas/oil–water interfaces are crucial to screening and evaluating surfactants. Great strides have been made in recent decades [5]. The experimental approach has been well established, and the workflow is as follows: a candidate surfactant is first selected. Then, studies on phase behavior and thermal stability are conducted. Subsequently, the surfactants with lower IFT measurements are adopted to perform adsorption and core flooding tests. An applicable surfactant should have the following features: good thermal stability under reservoir conditions (i.e., high temperatures and high pressures), being capable of reducing the IFT to 10^−2^ mN/m, low retention on the surface of reservoir rock, salt tolerance at reservoir salinity, and availability with an acceptable cost [22]. However, the experiments are very complicated since the chemicals are always mixed with some impurities, which will interfere with the analyzed results. Realizing high temperatures and high pressures is very challenging in the laboratory. It is noteworthy that the macroscopic properties (e.g., IFT and viscosity) are majorly determined by the molecular arrangement and kinetic behaviors of the molecules from a nanoscale point of view. The conventional analysis process of indoor tests always lacks microscopic information due to the deficiency of temporal–spatial resolutions in experimental facilities, and it can hardly capture detailed pictures of molecular motions and the evolution of the system from a microscopic perspective. In contrast, molecular dynamics (MD) simulation is extremely powerful in modeling the interactions between different molecules, even under harsh conditions [33,34]. It is a valuable complement to experimental and analytical approaches, which can provide profound perceptions of evolution processes with simulation time for microscopic systems and facilitate in-depth post-analysis [35,36]. As shown in Figure 5, by solving Newton’s second law regarding all the particles in the simulated systems, the trajectory profile (i.e., coordinates and instantaneous velocities at each step) can be derived. Then, the thermodynamics and kinetic properties can be predicted using the statistical mechanics method [37]. By correlating with experimental work, MD simulations can effectively reveal microscopic mechanisms that experiments cannot explain solely. Furthermore, it can also vividly visualize the evolution process of molecular configurations from a molecular point of view [36]. 

**Figure 6:**
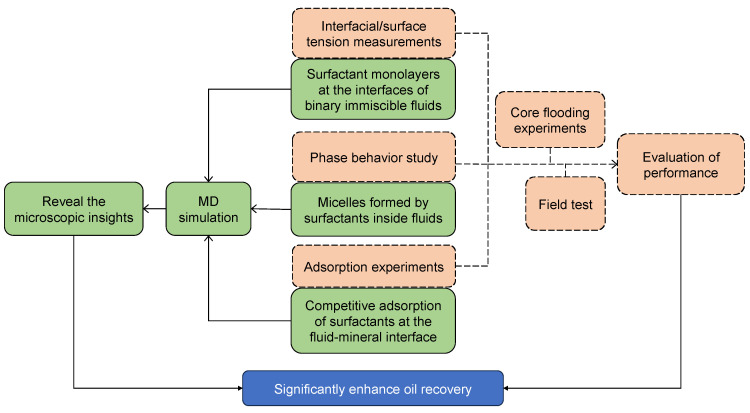
Relationships between molecular dynamics simulations and traditional experimental approaches to screening and evaluating the surfactants in enhanced oil recovery.

In recent years, with the rapid development of computer hardware, parallel computing, and graphics processing units (GPU) acceleration technology, as well as the advancement of theoretical and computational chemistry, the MD simulation method has been extensively employed in the research fields of chemical engineering and petroleum engineering [36,38,39,40,41]. Figure 6 shows the relationships between MD simulations and experimental work. Similar to experiments, MD studies can be categorized into three aspects. (1) Investigation of molecular configurations of surfactant-formed monolayers at the interfaces between two immiscible liquids, which corresponds to IFT measurements that are derived from experiments. It is increasingly being recognized by the interfacial science community that the choice of proper surfactants requires a fundamental understanding of both dynamic and static aspects of the IFT changes that occur in the presence of added surfactants. Using the MD simulation method, the factors that influence the interfacial performance of the surfactants with different structures can be well clarified [33,42]. At the same time, the interfacial configurations with molecular views (nanoscale) can be easily associated with the interfacial properties (macroscopic scale). (2) In aqueous solutions, surfactants with higher concentrations undergo self-assembly behavior and tend to form organized aggregates of large numbers of molecules, which are termed micelles [43]. (Note: the formation of micelles and their properties are out of the scope of this review.) The specific value of the threshold is termed the critical micelle concentration (CMC). Perturbation of the liquid–liquid interface is crucial in forming and breaking oil-in-water and water-in-oil microemulsions. The desired perturbation can be accomplished using surfactants in emulsifying or demulsifying formulations [44]. This situation corresponds to the experimental study of phase behaviors. (3) Investigation of interactions between the added surfactants and mineral surface, which corresponds to the adsorption test in the laboratory [13]. Understanding the retention and adsorption of polymers and surfactants in porous media is of key importance for designing viable EOR processes. These studies not only complement the experimental evaluation process of surfactant performance but also unravel the microscopic mechanisms for oil displacement. The MD simulation results can provide a theoretical basis and meaningful guidance for designing suitable surfactant formulations for specific reservoir conditions [45]. The first aspect (i.e., surfactant monolayers at the interfaces of binary immiscible fluids) will be the main focus of this review. 

**Figure 7:**
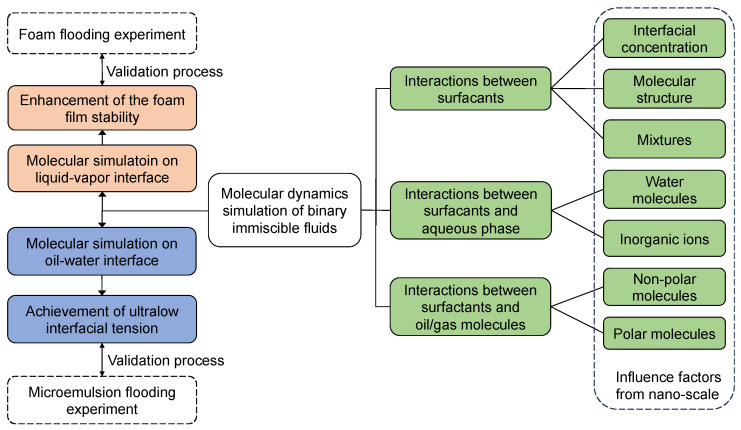
Computational schemes for molecular dynamics simulation of binary immiscible fluids.

The interfaces of two immiscible fluids can be divided into liquid–vapor interface (i.e., foam system) and oil–water interface (i.e., emulsion system) depending on the fluid’s phase, as shown in Figure 7 (in this review, the water phase denotes the aqueous/solution phase in general). The objectives are the improvement of foam film stability and the realization of ultra-low IFT for oil displacement under reservoir conditions, respectively. The interactions between (1) the surfactant molecules, (2) the headgroups of surfactants and water molecules, and (3) the surfactant alkyl tails and the molecules in the hydrophobic phase are very crucial to the interfacial performance of the selected surfactants. The combined effects of these three interactions determine how well the surfactants perform at the interfaces. 

The initial setup of the interfacial systems in MD simulations often employs the following geometry: a slab of liquid is positioned between two slabs of vapor (or another liquid) along the *Z*-axis, which is called the sandwich model (it consists of two phases). Under these circumstances, two interfaces parallel to the *X-Y* plane will be spontaneously generated. Each bulk phase is bounded by two independent interfaces. Using periodic boundary conditions allows for conditions representative of essentially infinite interfaces. The dimension of the simulation box can be determined by specifying the number of molecules desired and the expected phase density of each slab, whose values can be derived from experimental work. It should be noted that the lateral length (along the *Z*-axis) of the simulation box should be sufficiently greater than the size of the interfacial area (along the *X-Y* plane). The isobaric–isothermal–isointerface area ensemble (i.e., NPnAT) is recommended for the MD simulation. In this ensemble, normal pressure (perpendicular to the interface, also called bulk pressure) is maintained constant by adjusting the lateral length (i.e., *L_Z_*) of the simulation box. Meanwhile, the size of the interfacial area is also invariable during the simulation process. It is suggested to examine the potential energy of the systems and IFT values to identify whether the systems reach an equilibrium state. Note: The equilibration process at the water-surfactant-oil interface may require several microseconds due to the aggregation behaviors of the surfactants, which are much longer than previously reported simulated times [46]. This paper reviews the recent applications and advancements of the MD simulation method for surfactants in petroleum production and geological storage systems. It summarizes the surfactants’ interfacial performance at various interfaces and discusses the factors that influence the resultant interfacial properties in subsequent sections. 

## 2. Research Progress 

### 2.1. Interfacial Properties of the Surfactant-Formed Monolayers

#### 2.1.1. Evaluation Method for Interfacial Properties 

The interfacial properties of the surfactant-formed monolayers consist of the IFT, surface pressure–area (Π−A) isotherms, interface formation energy, and interfacial elasticity. MD simulation enables the efficient generation of diverse molecular models for various surfactants, allowing the computation of the systems’ thermal dynamics and kinetic properties within a condensed timeframe. When the interfaces are perpendicular to the Z-axis, the IFT can be calculated using a microscopic stress tensor, which is derived from the equation below [47]:

(4) $$\gamma =\int_{-\infty }^{+\infty }[P_{N}(z)-P_{T}(z)]dz$$

where γ represents the IFT, $P_{N}(z)$ is the bulk pressure (i.e., $P_{ZZ}$, also called normal pressure), and $P_{T}(z)$ is tangential pressure (i.e., $P_{XX}$ and $P_{YY}$, also called lateral pressure). The integral is defined over the boundary layer and can be extended to infinity. Note: If we consider a localized area of a specific geometry, the equation can be applied to a nonplanar surface (such as a spherical shape) [48]. The pressure tensor method is the predominant approach for determining the IFT for pure fluids and fluid mixtures. The basis of this method is to calculate the components of the diagonal element of the inhomogeneous pressure tensor $P_{kk}(z)$ using the Irving-Kirkwood (IK) formulation [49], which then feeds into Equation (4) to be employed to predict the IFT. In the IK method, the pressure tensor element $P_{kk}(z)$ is given by the expression below [50]:

(5) $$P_{kk}\left(z\right)=k_{B}T_{\rho }(z)+\frac{1}{A}\langle \sum_{i}^{N-1}\sum_{j>i}^{N}\frac{1}{\left|z_{i}-z_{j}\right|}{\left(f_{ij}(z)\right)}_{k}{\left(r_{ij}\right)}_{k}\rangle$$

where the subscript *kk* denotes the spatial coordinate, either *X*, *Y*, or *Z*. $k_{B}$ is Boltzmann’s constant, *T* is the absolute temperature, *A* is the interfacial area, *N* is the number of molecules, and the double sum involves the force on molecule *i* due to molecule *j*. $f_{ij}$ is the force on molecule *i* due to molecule *j*, and $r_{ij}$ represents the distance between molecules *i* and *j*. 

The prediction of interfacial elasticity can be used to evaluate the resistance to mechanical disturbance applied to the monolayers at the interface. Equation (3) can be written as follows [19]:

(6) $$E=\frac{d\gamma }{dlnA}=\frac{<\gamma_{i+1}-\gamma_{i}>}{<{SAPM}_{i+1}-{SAPM}_{i}>}$$

where $\gamma_{i}$ and ${SAPM}_{i}$ represent the IFT value and surface area per molecule for the surfactant at the *i*th concentration, and <…> means the statistical average. It offers the benefit of studying changes in surfactant behaviors with increasing or decreasing concentration while also evaluating the interfacial elasticity of the monolayers. Admittedly, the experimental measurements are dependent on the frequency of perturbation. When the frequency is extremely low (e.g., 0.1 Hz), the surfactant molecules in solutions can rapidly move to the interface, leading to considerable uncertainty in the measurement. According to our experience [19], past simulation work [21], and experiments [51], the data at ~10 Hz can be reasonably reproduced by MD simulation studies. 

**Figure 8:**
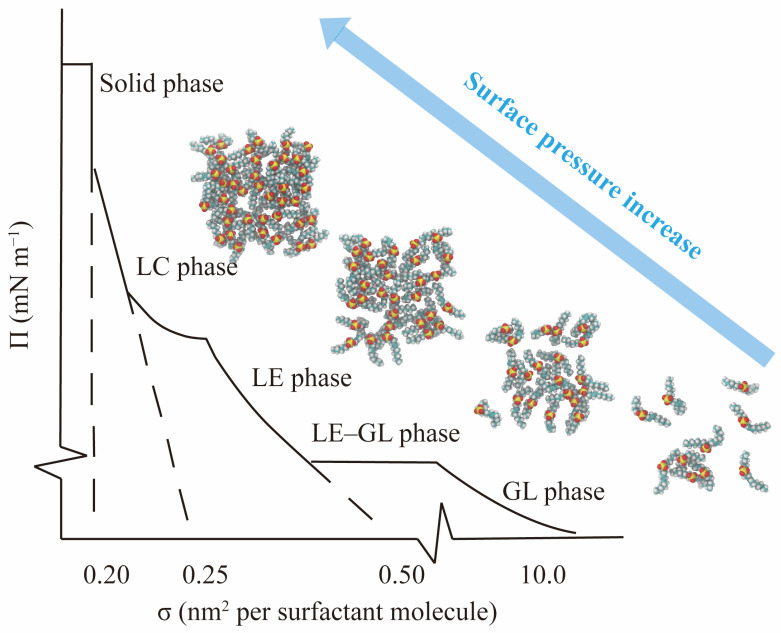
A schematic diagram of surface pressure (Π−A) isotherms as a function of the surface area per molecule of the surfactant monolayer. The molecular model of internal olefin sulfonate is used here. GL, LE, and LC mean gas-like, liquid-expanded, and liquid-condensed, respectively.

The monolayers are typically characterized in experiments by surface pressure–area (Π−A) isotherms, defined as a measurement at a constant temperature of surface pressure as a function of the available area for each molecule in the monolayers. The surface pressure of the monolayers can be calculated from the interfacial tension according to the following equation [20]:

(7) $$\Pi_{surface}(A)=\gamma_{0}-\gamma$$

where *A* is the area per surfactant molecule. $\gamma_{0}$ is the IFT of the pure gas/oil–water interfaces, and γ is the IFT of the interfaces with the surfactant monolayer. As shown in Figure 8, the curves of the Π−A isotherms can be generally classified into different monolayer phases (gas-like phase, liquid-expanded phase, and liquid-condensed phase) with different slopes, and the slopes become sharper as the monolayer becomes more compact. 

The interface formation energy (*IFE*) can be calculated to evaluate the stability of the interfaces and find the most probable interfacial concentration. The minimum IFT value always corresponds to the lowest *IFE* value. The parameter can be obtained from the following equation [52]:

(8) $$IFE=\frac{E_{total}-(n\times E_{surfactant,single}+E_{gas/oil–water})}{n}$$

where $E_{total}$ denotes the total energy of the entire system. $E_{surfactant,single}$ denotes the energy of a single surfactant molecule calculated from a separate MD simulation in a vacuum at the same temperature. $E_{gas/oil–water}$ denotes the energy of a pure gas/oil–water system obtained from a separate MD simulation with the same number of molecules (gas/oil/water) used in the total system at the same temperature. n is the total number of surfactant molecules. The lower IFE values indicate that inserting an extra surfactant molecule requires less energy to go into the interfaces. 

#### 2.1.2. Effect of Interfacial Concentration and Molecular Structure

**Figure 9:**
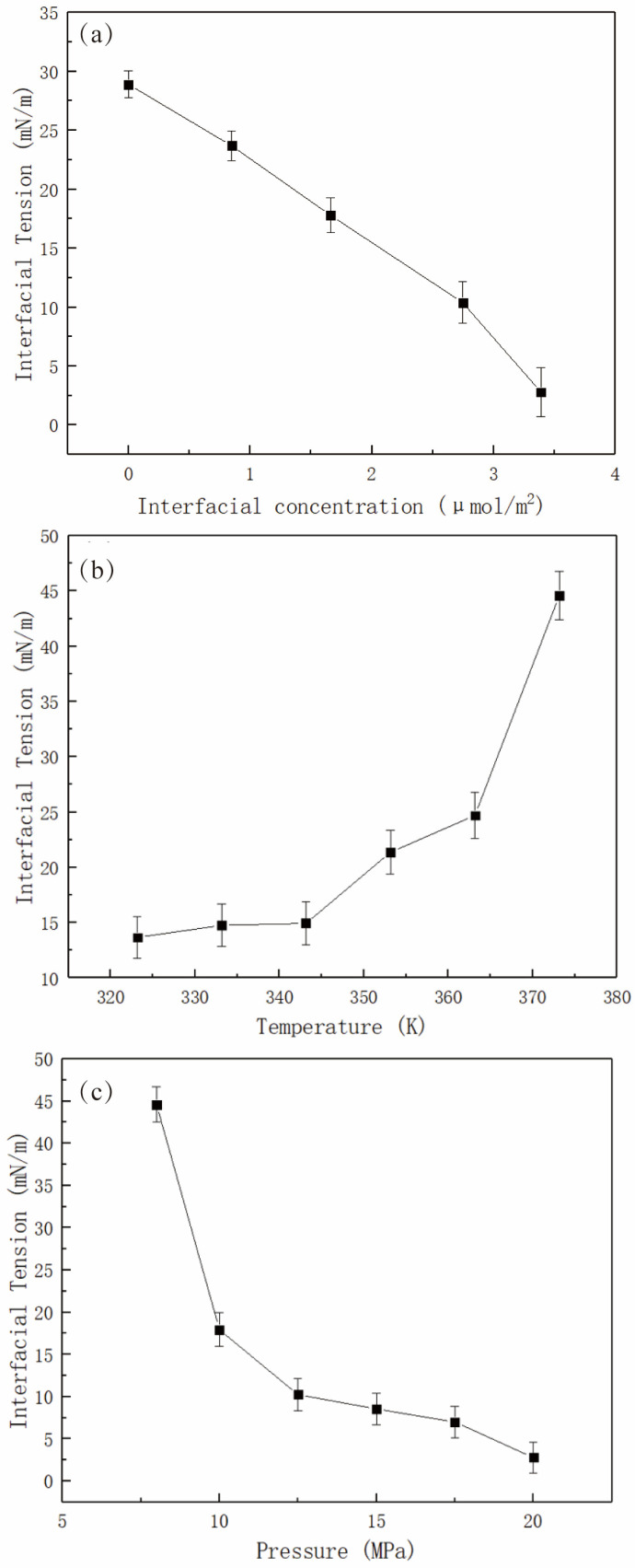
The influencing factors affect the static stability (i.e., IFT values) of SDS-stabilized CO_2_ foam films. The effects of (**a**) interfacial concentration, (**b**) temperature, and (**c**) pressure on the IFT values at the CO_2_–water interfaces [16].

With increasing surfactant concentration, the IFT between a fluid (e.g., oil) and the surfactant aqueous solution decreases linearly below a specific concentration, showing an inflection point that can be regarded as the CMC [17,53]. In practice, the CMC can be employed to indicate a concentration of the solution at which the interface has been entirely covered by the surfactant molecules (i.e., saturation coverage). The MD simulation studies can reproduce the linear relationship well [16,19,26]. Please note that the interfacial concentration (i.e., an interfacial area that is occupied by each surfactant molecule) is used in the simulation studies since the bulk phase (in nanoscale) in the simulation is much smaller than that in the experimental cases (in millimeter scale). The MD simulation study can also characterize the saturation coverage at the interfaces. In summary, CMC is a useful concept to link simulations and experiments. However, to directly determine the CMC, it is essential to establish a correlation between the interfacial concentration and the concentration in the bulk solution. Fan and coworkers [16] have investigated the effect of interfacial concentration, temperature, and pressure on the static stability (i.e., IFT) of the sodium dodecyl sulfate (SDS)-stabilized CO_2_ foam film system. Figure 9 shows that as the interfacial concentration of surfactant rises, the IFT values fall linearly. The results can be well understood as SDS molecules form a dense and thick monolayer under high-concentration conditions, preventing CO_2_ and water molecules from contacting each other. Noteworthily, the linear relationship can correspond well with experimental results [53]. Doing a series of MD simulations at different interfacial concentrations helps us find the saturation coverage of the surfactant at the CO_2_–water interface. At higher concentrations, the IFT may become negative (due to a curved interface) [26] or show an inflection point (due to the formation of micelles near the interface) [19]. Then, the concentration of saturation coverage was used in the subsequent studies (i.e., the effect of temperature and pressure on the interfacial properties). Low temperatures and high pressures are favorable conditions for reducing IFT values due to the enhancement of interactions between the CO_2_ molecules and the surfactant alkyl tails, thus inhibiting the interactions between CO_2_ and water molecules. 

**Figure 10:**
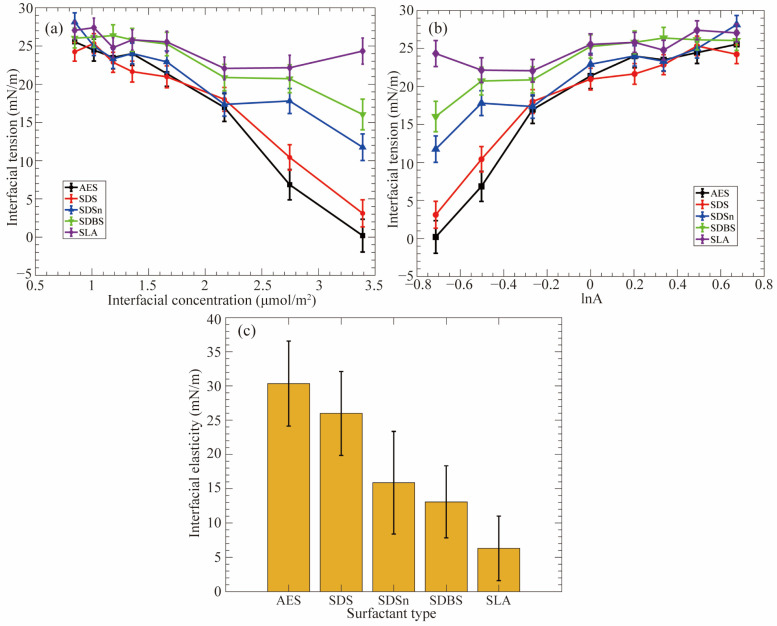
Interfacial properties of the surfactant-formed monolayers at the CO_2_–water interfaces. (**a**) Variation of the IFT values with the increase in the interfacial concentration; (**b**) Plotting of the IFT versus the natural logarithm of the area per surfactant molecule; (**c**) The predicted interfacial elasticity [19].

The architecture of surfactant molecules has a significant impact on the interfacial properties. Jia and coworkers [19] have investigated the effects of the molecular structure of surfactants on the dynamic stability (i.e., interfacial elasticity) at the CO_2_–water interfaces. According to the data presented in Figure 10, the ranking of CO_2_ foam stability enhancement capacity is as follows: sodium polyoxyethylene alkyl ether sulfate (AES) > SDS > sodium decyl sulfonate (SDSn) > sodium dodecylbenzene sulfonate (SDBS) > sodium laurate (SLA), which aligns well with the results obtained from experiments. Using the MD simulation method, the researchers have discovered that lnA = 0 (note: A denotes the interfacial area per surfactant molecule, and here it is equal to 1 nm^2^/molecule) serves as a critical point. When the interfacial concentration is low (i.e., A is larger than 1 nm^2^/molecule, and lnA > 0), the difference in IFT variations between different surfactant monolayers is insignificant. Thus, the interfacial elasticities of the monolayers are similar. In contrast, when the interfacial concentration is high (i.e., A is less than 1 nm^2^/molecule, and lnA < 0), the impact of molecular structure on the reduction of IFT becomes increasingly evident with the concentration increasing. The IFT variations of the monolayers distinctly vary, and the difference in interfacial elasticity of various monolayers is very clear. Consequently, this part (i.e., lnA < 0) plays a vital role in determining the interfacial elasticity of the monolayers. Figure 10c illustrates the predicted interfacial elasticity using data points in the range of lnA < 0. Additionally, due to the occurrence of EO chains in the AES surfactant, the carbon tails are adequately solvated by the CO_2_ phase while maintaining a balanced interaction between the headgroup and the water phase, thus significantly improving the hydrophilic–CO_2_-philic balance (HCB). Though the SDBS surfactant exhibits favorable features (i.e., large interfacial width and a high degree of interfacial coverage) as an EOR chemical agent, its performance at the CO_2_–water interface is very limited since the presence of the phenyl group makes SDBS surfactant too hydrophobic to a CO_2_ phase (thus a poor HCB). However, these factors are very beneficial to the IFT reduction at the oil–water interface. 

**Figure 11:**
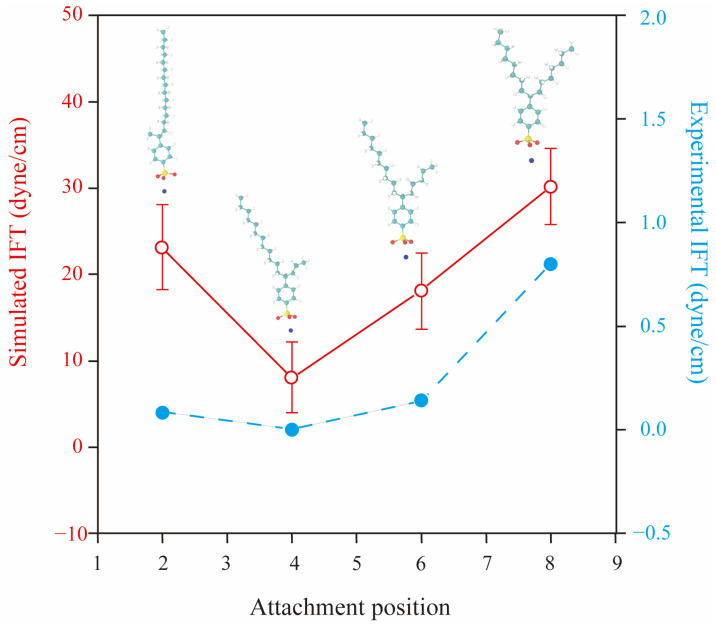
Variations in the interfacial tension (IFT) at the oil–water interface depend on the attachment position of the benzene sulfonate groups. The solid red line, open circles indicate the IFT results obtained from MD simulations. The dashed blue line and solid circles are IFT results from experiments (adapted from [52]).

Linear alkylbenzene sulfonates (LAS) possess relatively simple headgroups and several hydrophobic tails with diverse structures. Thus, many MD simulations have chosen LAS as a research object to study the influence of the molecular structure of surfactants on their interfacial properties [35,52,54,55,56,57]. Chen et al. [56] discovered that adding a certain length of the branched structure to SDSn could make the performance better at the air–water interface. Zhao et al. [57] found that among the sodium hexadecane benzene sulfonate (SHBS) isomers, SHBS-1C16 with a benzene ring on the first carbon atom has stronger intermolecular interactions and greater electrostatic repulsion between the headgroups due to its long single-chain tail, which results in a disordered interfacial structure at the air–water interface. In contrast, SHBS-5C16, with the benzene ring on the 5th carbon atom, has two hydrophobic tails with different lengths. The steric hindrance effect of the short tail can inhibit the aggregation behavior of the surfactant molecules, thus improving the stability of the monolayers. As shown in Figure 11, Jang et al. [52] further proved that the twin-tailed structure of SHBS-4C16 holds the best interfacial performance at the decane–water interface. It has the lowest IFT and the lowest interfacial formation energy. Meanwhile, the formed monolayer has the largest interfacial width, and the molecules are closely distributed inside the monolayer. He et al. [55] conducted 36 simulations on the adsorption of LAS-mCn (*m* = 1~6, *n* = 1~11) with different carbon tail lengths and different benzene ring attachment positions at the air–water interface. They pointed out that LAS with short carbon tails have high solubility and flowability in the aqueous phase, and their excessive hydrophilicity prevents adsorption at the interface. LAS-1Cn possesses a single-tailed structure and is apt to aggregate at the interface, which deteriorates the structural integrity of the formed monolayers. Only LAS with a high degree of branchedness and long carbon tails can cover the interface and minimize the IFT at the interface to the fullest extent. In addition, it has been found that the salt tolerance of twin-tailed SHBS-5C16 is better than that of single-tailed SHBS-1C16 [54], and the salt tolerance of SDBS isomers is 6C12 > 4C12 > 1C12 [35]. Concerning the nonionic surfactant, take C12E3 (i.e., triethyleneglycol 1-dodecyl ether) and C6C5CE3 (i.e., triethyleneglycol 6-dodecyl ether) as examples. The twin-tailed C6C5CE3 has better salt tolerance than the single-tailed C12E3 at the water–dodecane interfaces [58]. (We will further discuss the effect of molecular architecture and counterions in the solutions in Section 2.2 and Section 2.3) In summary, the twin-tailed structure cannot only lead to lower IFT but also achieve better salt resistance compared with a single-tailed structure. 

**Figure 12:**
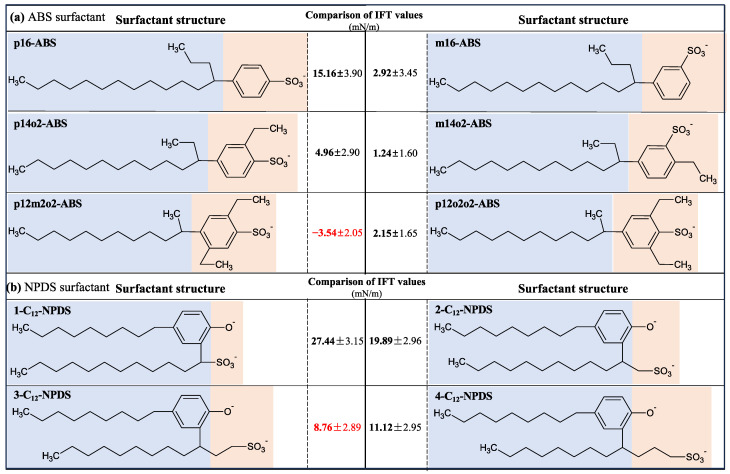
Effect of the headgroup architecture of the surfactants on the interfacial performance at various interfaces. The red bold values indicate the minimum IFT. The effect of headgroup size on the IFT values of (**a**) ABS surfactants at the decane–water interfaces and (**b**) NPDS surfactants at the air–water interfaces (adapted from [65,66,67]).

Moreover, Adkins and coworkers [59] showed that introducing extra-weak hydrophilic radicals, such as hydroxyl and ethoxy groups, into the molecular chain of the surfactants could also enhance their interfacial activities. Hou and coworkers [60,61] found that the interaction of oligomeric surfactants with water molecules and oil molecules is stronger than that of single-chain and dimer-typed surfactants, thus leading to the lowest IFT at the oil–water interface. Shi and coworkers [62] demonstrated that the Gemini surfactants, which utilize a linker group to associate two monomers, were more effective in reducing the IFT than the typical monomolecular surfactants at the oil–water interface. Han et al. [63] found that Gemini surfactants with shorter spacers exhibit better surface activity. In comparison, longer spacers bind more oil molecules to the carbon chain, reducing the surface activity. Wang et al. [64] demonstrated that the self-assembled morphologies of Gemini surfactants change with the decrease in the spacer length. Tan and coworkers [65] investigated the effect of headgroup size on the interfacial performance of six isomers of alkyl benzene sulfonate (ABS) and found that the IFT at the decane–water interface gradually decreased with the increase of the number of substituent groups in the benzene ring structure and the increase of headgroup size to some extent (see Figure 12a). However, Gao and coworkers [66,67] showed that the interfacial performance is not necessarily better when the headgroup size is larger. As shown in Figure 12b, the IFT of nonylphenol-substituted dodecyl sulfonates (NPDS) at the air–water interface decreases and then rises with the increase in headgroup size, and 3-C12-NPDS has the lowest IFT and IFE. Consequently, the interfacial properties are the combined effect of changes in molecular chain structure, attached chemical groups, and the amount of surfactants (i.e., interfacial concentration) at the interfaces. 

#### 2.1.3. Synergistic Effect of Surfactant Mixtures 

In practical application, a single surfactant usually cannot fully meet complex reservoir conditions, such as temperature, pressure, and salinity. Thus, it is suggested that a variety of surfactants be chosen at the same time [68,69]. The synergistic effect of the surfactant mixtures can significantly improve the interfacial performance compared with the single surfactant at the interfaces [5]. The adsorption behavior of mixed surfactants at the gas/oil–water interfaces with varying molar ratios was studied using MD simulations. The researchers showed that, as compared to pure surfactants, the monolayer formed by the adsorption of their mixture is more compact, thus leading to better interfacial activities. The synergistic effects of ionic surfactants are mainly due to the strong electrostatic interactions between anionic and cationic headgroups, which shield the electrostatic repulsion between the same electrically charged headgroups and lead to a smaller separation distance between the surfactant molecules (i.e., closely packed) at the interface [70,71]. The combination of anionic and cationic binary surfactant mixtures can lower the CMC value and reduce the IFT compared with individual surfactants. At low concentrations, surfactants with opposite charges pack as co-surfactants like Gemini, while at high concentrations, anionic and cationic surfactant mixtures generate closely packed adsorption layers at the interfaces with strong viscoelasticity and negligible diffusion exchange between the interface and bulk solutions. 

**Figure 13:**
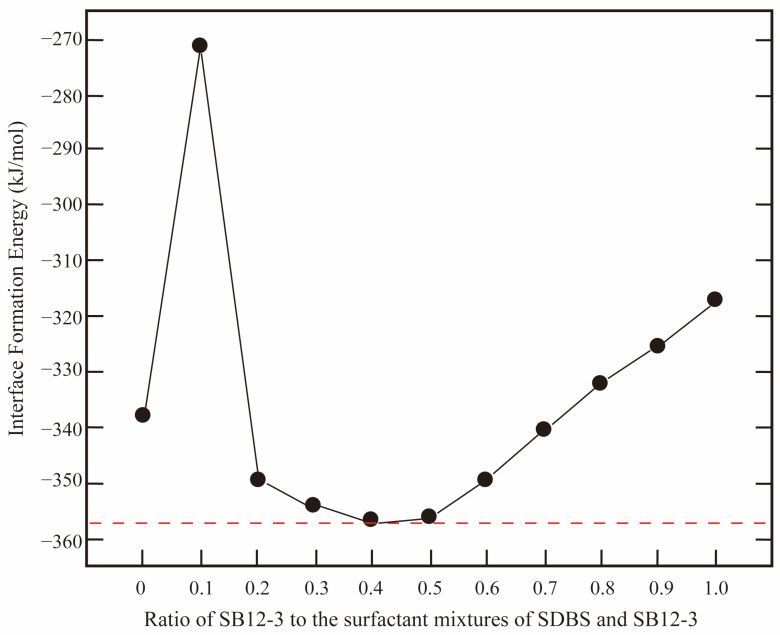
Ratio effect of the individual surfactant in the surfactant mixtures on the interface formation energy. The black solid line and solid circles are calculated interface formation energy (IFE) from MD simulations. The red dashed line indicates the minimum IFE [73,74].

However, Agneta et al. [72] reported that antagonism exists between the anionic and cationic surfactant mixtures under high salinity conditions at the gas–water interfaces. In contrast, strong synergism exists between the anionic/cationic and nonionic binary surfactant mixtures. Compared with anionic/cationic surfactants, zwitterionic surfactants simultaneously have both positive and negative electrically charged headgroups. Due to the large size of the headgroups, the surfactant molecules are apt to lie flat at the interface, leading to a disordered arrangement of the molecules and a loose monolayer. Wang et al. [21] found that the introduction of an appropriate amount of lauryl betaine (LB-12) could significantly improve the interfacial performance of sodium *α*-olefin sulfonate (AOS-14). When the ratio of AOS-14 to LB-12 equals 7:3 at the interface, the interfacial elasticity is the largest, and the binding energy is the lowest, indicating that the monolayer is the most stable. Its resistance to external perturbation is the strongest (the molecular insights are discussed in Section 2.2.3). Li et al. [73,74] found that the surfactant mixtures of dodecyl sulfonate betaine (SB12-3) and SDBS have the most stable interface (i.e., the lowest IFT) when the ratio of SB12-3 to SDBS equals 4:6. As shown in Figure 13, with the increase in the fraction of SB12-3, the IFE decreases at the beginning and then rises from 50% concentrations. Gao et al. [75] reported that the presence of LB-12 surfactant can further improve the stability of alkyl polyoxyethylene carboxylate (AEC)-stabilized foam film. LB-12 can modulate the ordering of AEC at the air–water interface, and the electrostatic structure becomes denser with the increasing concentration of LB-12. In addition to well reproducing the interfacial properties of various surfactants, we can also effectively investigate the diffusion and aggregate behaviors of the surfactant molecules in the monolayers at the interface (Section 2.2), surfactant headgroups–aqueous phase interactions (Section 2.3), and surfactant alkyl tails–hydrophobic phase interactions (Section 2.4) in the vicinity of the interfaces using MD simulation method. 

### 2.2. Molecular Views of the Interfacial Structure 

#### 2.2.1. Characterization of the Microstructure at the Interface

The interfacial properties are mainly determined by the microstructure at the interfaces. MD simulation method can straightforwardly and quantitatively study the microstructure of the intermediate regions (i.e., interfaces) and surfactant behaviors within these regions. Furthermore, they can effectively correlate molecular configurations at nanoscale and interfacial properties measured in the laboratory. The microstructure of the interfaces can be characterized by the mass density distribution of different components along the *Z* direction that is normal to the interface. The density profile of the *i*th component (without excess absorption at the interface) obtained from the simulations can be fitted using the following hyperbolic tangent function [52] as below:

(9) $$\rho_{i}\left(z\right)={0.5\rho }_{i,bulk}-0.5\rho_{i,bulk}tanh\left[\frac{2(z-z_{c})}{d}\right]$$

where $\rho_{i}$ is the density of the *i*th component, $z_{c}$ is the position of the Gibbs dividing surface, and *d* is the adjustable parameter related to the interfacial width. Furthermore, the distribution of surfactant headgroups at the interfaces can be well fitted by a Gaussian function [55], which can be expressed by the equation as follows:

(10) $$\rho (z)=\frac{N_{s}}{\sigma \sqrt{2\pi }}exp\left[-\frac{{\left(z-z_{p}\right)}^{2}}{2\sigma^{2}}\right]$$

where $N_{s}$ is a constant that, in fact, refers to the number of atoms in each monolayer peak, $z_{p}$ is the position of the peak center, and σ is the standard deviation, which shows the width of each peak. 

By analyzing the distribution patterns of the mass density of the individuals, we can obtain interfacial width at various interfaces, though there are different criteria [52,67,68,69,70,71,72,73,74,75,76]. A common practice for defining the interfacial width for the liquid–vapor interface is the “10–90” criterion [66,77], which is the distance between two positions where the density varies from 10% to 90% of the density of the bulk phase. However, it becomes more complicated when surfactants are introduced at the liquid–liquid (e.g., oil–water) interfaces due to the presence of two sub-interfaces. In this case, the “90–90” criterion is suggested [52,76], which is the distance between two positions where the densities of water and oil are 90% of their bulk density. The interfacial width generally monotonically increases with the number of surfactant molecules at the interface since the surfactant molecules become more tightly packed and the carbon tails become more vertically oriented in relation to the interface [26]. When the interface is no longer flat, the definition of the interfacial width breaks down and no longer reflects the actual interfacial width. However, it does reflect the size of the undulations. 

The interfacial coverage indicates the degree of integrity (i.e., the fraction of coverage) of the monolayers at the interface, which can also be quantitatively characterized by the following equation [26]:

(11) $$\varphi =\frac{{N_{0}-N}_{s}}{N_{0}}\times 100\%$$

where φ denotes the interfacial coverage, and *N* is the number of water molecules that are within 0.5 nm (an empirical parameter for the estimation) from the gas/oil phase. Subscripts 0 and *S* denote the pure oil/gas–water system and systems containing surfactants, respectively. According to the molecular capillary wave theory, the variations of IFT values at the oil–water interfaces are inversely proportional to the interfacial width [26,78], whereas the IFT values at the gas–water interfaces are influenced by both interfacial width and interfacial coverage [18]. 

The order parameter can be employed to assess the ordering degree of the surfactant alkyl tails in relation to the *X*, *Y*, and *Z* axes at the interfaces and can be defined as follows [26]:

(12) $$S_{CH}=\frac{1}{2}\langle 3{cos}^{2}\theta -1\rangle$$

the order parameter $S_{CH}$ characterizes the orientations of the segments/vectors (pointing from carbon atoms *i* − 1 to *i* + 1) at the interface, θ represents the angle between the segments/vectors and the axes. <…> means ensemble average. If $S_{CH}$ is equal to zero, it signifies that the orientation of the segments/vectors is disordered. When the values approach 1 or −0.5, it indicates that the alkyl tails tend to align perpendicular to or parallel to the interface. A complementary analysis of the inclined angle of the alkyl chains helps evaluate the overall trend of the tail orientation of a given alkyl chain, which is accomplished by creating a vector between the base of the chain (the first carbon atom that is next to the headgroup) and the terminal carbon atom. With this vector defined, we can take the projection on the monolayer normal to determine the degree of inclination.

Finally, utilizing the radial distribution function (RDF) allows for the characterization of the average radial packing of atoms within a given system. It is expressed as follows [19]:

(13) $$g(r)=\frac{n(r)}{4\pi \rho r^{2}∆r}$$

where g(r) is the RDF, n(r) is the average number of atoms in a shell of width ∆r at a distance r from the reference atom. ρ is the average atom density. The presence of peaks identified at long ranges indicates a high degree of ordering. It can characterize the typical arrangement of particles at the interface and in the bulk phase. Furthermore, it can also be used to estimate the potential of mean force (PMF). 

#### 2.2.2. Effect of Interfacial Concentration on the Packing State of the Surfactants

**Figure 14:**
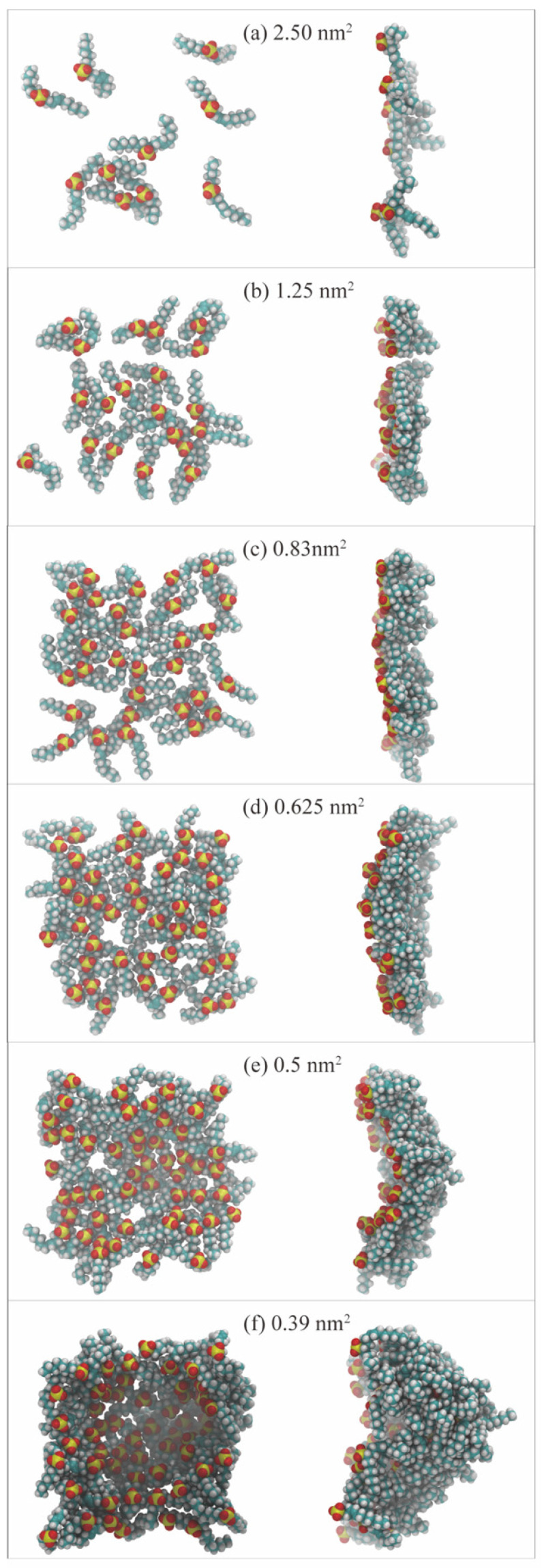
Final molecular configurations of IOS surfactant monolayers at the decane–water interface under the equilibrium state. Panels (**a**–**f**) indicate the conditions with different interfacial concentrations. The left column is front view, and the right column is side view (adapted from [26]).

The interfacial concentration, a crucial factor, plays a significant role in modulating the spontaneous organization of surfactant molecules. Increasing the concentration of surfactants triggers a process of self-assembly driven by noncovalent interactions between the molecules, leading to the formation of aggregates at the interface. The MD simulation method, a powerful tool, allows for a detailed study of the effect of interfacial concentration on the packing state of surfactants, particularly under extreme conditions. This method also provides a visual representation of the evolution process of the monolayers from an atomistic perspective, enabling a comprehensive analysis of the influence factors induced by molecular architecture. In the MD simulation method, surface area per molecule (SAPM) is preferred to describe the interfacial concentration of the surfactants. Given that the simulated system’s interfacial area (size of cross-section area) is invariable, SAPM values become smaller with the increase in surfactant molecule number (until it reaches saturation coverage) at the interfaces. Accordingly, the geometric configurations and interfacial properties of the surfactant-formed monolayers change. Figure 14 illustrates the variations of the monolayer’s morphology at the oil–water interface formed by internal olefin sulfonate (IOS) with the increase in interfacial concentration [26]. Based on SAPM values, the evolution process of the monolayers can be divided into four stages, as follows:

(1) When the number of surfactant molecules at the interface is very few, the SAPM value is large (2.5 and 1.25 nm^2^ per surfactant molecule), as shown in panels a and b. The separation distances between the molecules are relatively large. In this circumstance, the interaction force between each other can be negligible. This state is called the gas-like (GL) phase. Since the molecular arrangements of the monolayers are sparse and the resulting interfacial widths are small, the interfacial performance of the monolayer is poor, and many hydrocarbon molecules can directly contact water molecules at the intermediate region via the gap that the surfactant molecules are not occupying.
(2) As the number of surfactant molecules increases at the interface, SAPM values decrease, as shown in panels c and d, and the interaction force between each surfactant molecule is enhanced. This state is called the liquid-expanded (LE) phase. At this moment, the monolayers become denser than those in the GL phase, and the orientation angles of surfactant alkyl tails are randomly distributed toward the oil/gas phase. The void space that remains in the monolayers allows for continued interaction between oil/gas and water molecules still occurs.
(3) When the number of surfactant molecules reaches the saturation concentration at the interface, SAPM reaches the critical minimum point (0.5 nm^2^ per surfactant molecule), as shown in panel e. The molecular arrangement of the monolayers changes from a loosely packed pattern to a densely packed pattern, marking the transition to the liquid-condensed (LC) phase. In the LC phase, surfactant molecules are distributed close to each other, and most of the surfactant alkyl tails tend to be perpendicular to the interface. The absence of void space in the monolayers and the resulting largest interfacial widths allow for the best performance, effectively preventing the interactions and contacts between oil/gas and water molecules in the intermediate region.
(4) When the interfacial concentration exceeds the concentration of saturation coverage, the interface becomes visibly curved (a concave surface), as shown in panel f. The interface becomes unstable and can undergo mechanical buckling to increase the interfacial area so that excessive surfactant molecules can be adsorbed at the contact surface between the oil/gas and water phases. In this circumstance, some surfactant molecules in the monolayers can also escape from the interface and form stable 3D structures such as vesicles and bilayers. As a result, the stability of the monolayer can recover. The interfacial properties change to different degrees as the shape of the surfactant monolayers changes over time.

The MD simulation method gives detailed images of the dynamic evolution process of monolayers’ morphology with increasing interfacial concentration and enables quantitative characterization of phase transitions and structural change. Wei and coworkers [79] reported entropic changes in SDS surfactant for phase transitions, which are −29.7 J mol^−1^ K^−1^ for the transition from 2D GL film to 2D LE film and −42.0 J mol^−1^ K^−1^ for the transition from 2D LE state to 2D LC film. These values gave us an intuitive insight into these phase changes in the surfactant monolayer. MD results reveal that the change in the monolayers’ thickness associated with LC–LE transition is mainly due to a shortening of the surfactant alkyl tails, with little change in the average tilt angle of the headgroups [80]. Meanwhile, it has been observed that multiple phases can coexist within one monolayer [77]. We remark that the saturation coverage (LC phase) should be satisfied to maximize the interfacial performance of the surfactants. A comparative analysis of pertinent research findings [26] determined that monolayers composed of elongated single-chain molecules, such as AOS and SDS, exhibited interfacial buckling at elevated interfacial concentrations. By contrast, surfactant monolayers featuring twin-tailed structures, such as IOS, rhamnolipid, and DPPC, could achieve interface saturation and exhibit curved bucking at lower concentrations. This suggests that surfactants with twin-tailed structures may minimize or even eliminate cosolvent requirements (i.e., saving cost) and possess superior interfacial performance. However, it should be noted that SDS and DPPC differ in that SDS is a water-soluble surfactant while DPPC is not. 

#### 2.2.3. Effect of Molecular Structure and Synergism on Monolayers’ Morphology 

The type of surfactant headgroups and the architecture of surfactant alkyl tails can directly influence the diffusion behaviors of the surfactant molecules at the interface, thus affecting the corresponding interfacial properties. Tan et al. [65] conducted an in-depth study of the evolution of monolayer morphologies formed by six isomers of ABS surfactants at the decane–water interfaces. They showed that the GL–LE phase transition can be accelerated by disubstituted ABS surfactants while being delayed by trisubstituted ABS surfactants. Meanwhile, they found that large undulations are a sign of a collapse of the interface under extremely high surfactant concentrations. Shi and Guo [54] reported that the bending modulus can control the further transformation pathway from buckling to a protruding bud at the interface, which majorly depends on the tail length and interfacial surfactant coverage. They introduced area compressibility and bending modulus, and they showed that the bending modulus becomes larger as the tail length grows, indicating that the energy cost of bending the monolayer increases as the monolayer becomes thick. Likewise, Munusamy et al. [81] reported the segregation of molecular aggregates from the interface into the bulk water in the anionic rhamnolipid (Rha-C10-C10) monolayer at higher concentrations. In contrast, in the nonionic Rha-C10-C10 monolayer, the molecules are still distributed at the interface. Furthermore, the presence of a second rhamnose group can decrease the aggregate number [42]. These findings from MD simulations have deepened our understanding of the molecular architecture’s effect on the dynamic behaviors of the surfactants and the morphological evolution of the monolayers at the interfaces. 

**Figure 15:**
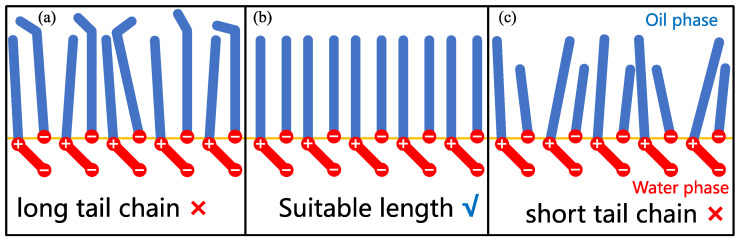
A schematic diagram of the interfacial structures formed by betaine (i.e., zwitterionic surfactants) and anionic surfactants with different carbon tail lengths. (**a**) Anionic surfactant has a longer carbon tail than betaine surfactant. (**b**) Anionic surfactant has a carbon tail with the same length to betaine surfactant. (**c**) Anionic surfactant has a shorter carbon tail than betaine surfactant. The headgroups and carbon tails of surfactants are indicated by red and blue colors, respectively. The oil–water interface is indicated by the yellow line.

The interactions between surfactant molecules in the monolayers are also subjected to multicomponent surfactant mixtures. As aforementioned, Wang et al. [21] found that the influence of LB surfactant on AOS surfactant is nonmonotonic with the change in ratio. The surface dilatational modulus (also known as interfacial elasticity) has a maximum when LB is 30% in the monolayer. They demonstrated that this overall impact is rooted in two competing effects as determined by MD simulation findings. They investigated the orientation of the headgroup of LB molecules. They found that it is tilted relative to the monolayer normal, and the tilt angle increases with increasing LB concentration at the interface. In contrast, the favorable interactions between the S (from AOS) and N (from LB) surfactant atoms (which can be demonstrated by the order parameters of the carbon tails) and the hydration of the carboxylate group of LB surfactant can inhibit the tendency of LB headgroups to become nearly parallel to the monolayer. Thus, the effective headgroup size (i.e., SAPM) is lower (compared to pure LB case) because favorable interactions between LB and AOS surfactants suppress the flexibility of the headgroup of LB. When the proportion of LB is higher than 70%, AOS–LB interactions are insufficient in constraining the headgroup orientation. The corresponding morphology can be depicted in Figure 15. As is observed, there are many gaps in the loose monolayer formed by zwitterionic surfactant (such as LB) molecules. The small headgroup size of ionic surfactants (such as AOS) can easily enter these gaps to prevent water and oil molecules from coming into contact with each other. The zwitterionic surfactant molecules (such as the carboxylate group in the headgroup of LB) penetrate the water phase to a greater extent, forming the “primary layer” in the monolayer, while most of the ionic surfactants occupy the gaps between the hydrophobic tails to form the “secondary layer” in the monolayer (reflecting the favorable interactions between the S and N atoms) [21,75,82,83]. The monolayers formed by the surfactant mixtures will perform best when the newly introduced surfactant has a suitable length of carbon tails in relation to the pre-existing surfactants. The matching of carbon tail lengths reflects the effect of van der Waals force interactions between the hydrophobic tails of different types of surfactants on the interfacial structure and properties [84]. 

When the components involved in the mixtures are molecules without ionic groups (i.e., cosolvent), such as octanol, decanol, dodecanol, and tetradecanol, the matching of carbon tail lengths becomes the dominant factor in determining the synergistic effects rather than the headgroup size and the electrical properties [39,40]. Surfactants with short alkyl chains have a higher tendency to transfer from the interface to the solution, which breaks down the tightly packed network at the interface. Ergin and coworkers [71] reported that the translational excess entropy due to the tail group interactions can discriminate between the synergistic system of SDS and LB-12 and the nonsynergistic system of SDS and cocamidopropyl betaine (CAPB). Therefore, we can use the MD simulation method to evaluate the synergistic effect of different surfactant mixtures. In addition, Jia and coworkers [85] investigated the interfacial assembly process and configuration of the pseudogemini surfactants consisting of SDBS and 4,4′-oxydianilinium chloride (ODC). They found that SDBS and ODC showed the vertical and horizontal arrangements at the oil–water interface, respectively, and the interfacial assembled configuration presented an unexpected “H” shape rather than the traditional “U” shape. They claimed that the cation–π interaction is responsible for the SDBS/ODC assembly mechanism and the final the oil–water interface configuration. In a word, the formation of closely packed and stable interfacial monolayers requires good compatibility between different surfactant (or cosolvent) molecules. 

### 2.3. Surfactant Headgroup Solvation and Counterion Effect in Aqueous Phase

Surfactant-formed monolayers possess an inherent electric charge on their surface, resulting in the presence of surface potential. Ions of opposite charge (counterions) are attracted to the surface, while those of like charge (co-ions) are repelled. An electric double layer (EDL), which is diffuse because of mixing caused by thermal motion, is thus formed [17]. The EDL can be described as consisting of two distinct layers: an inner layer that may contain adsorbed ions and a diffuse layer where ions are distributed according to the influence of electrical forces and thermal motion. Taking the surface electric potential to be $\psi^{0}$, and applying the Gouy-Chapman approximation, the electric potential ψ at a distance *x* from the surface is approximately predicted by the following equation:

(14) $$\psi =\psi^{0}exp(-3.288\sqrt{I}x)$$

where *I* is the ionic strength, given by $I=(1/2)\sum_{i}c_{i}z_{i}^{2}$, where $c_{i}$ is the concentration of ions and $z_{i}$ is the charge number of ions. 

The presence of charge at crude oil–aqueous contacts may arise from the ionization of surface acid functionalities. The presence of charge at gas–aqueous interfaces may arise from the adsorption of surfactant ions. When surfactant molecules adhere to interfaces, they have the potential to modify the surface electric charge, therefore influencing the concentration of inorganic ions in the vicinity. Additionally, it is worth noting that the headgroups of surfactants can create hydrogen bonds with water molecules. The adsorption and aggregation phenomena of water molecules and inorganic salt ions (i.e., counterions) close to the headgroups of surfactants can potentially influence the interfacial structure of surfactant monolayers, causing alterations in macroscopic characteristics. Using the MD simulation method, it becomes possible to engage in comprehensive analyses of the hydrogen bonding interactions occurring between the headgroups and the surrounding water molecules. Additionally, these simulations allow for the examination of the electrostatic interactions that take place between the headgroups and the inorganic salt ions.

#### 2.3.1. Hydration Shell Structure and Hydrogen Bonding

**Figure 16:**
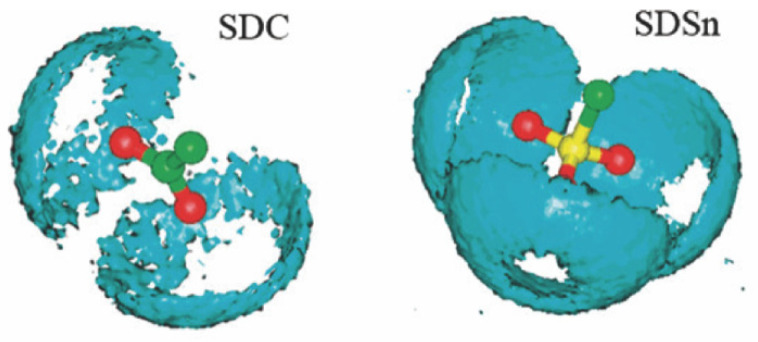
The spatial distribution function of water molecules surrounding the carboxylic acid group (**left side**) and sulfonate group (**right side**), respectively. Oxygen atoms are represented by red balls, the sulfur atom is indicated by the yellow ball, carbon atoms are indicated by green balls, and water molecules are indicated by cyan shading [86].

**Figure 17:**
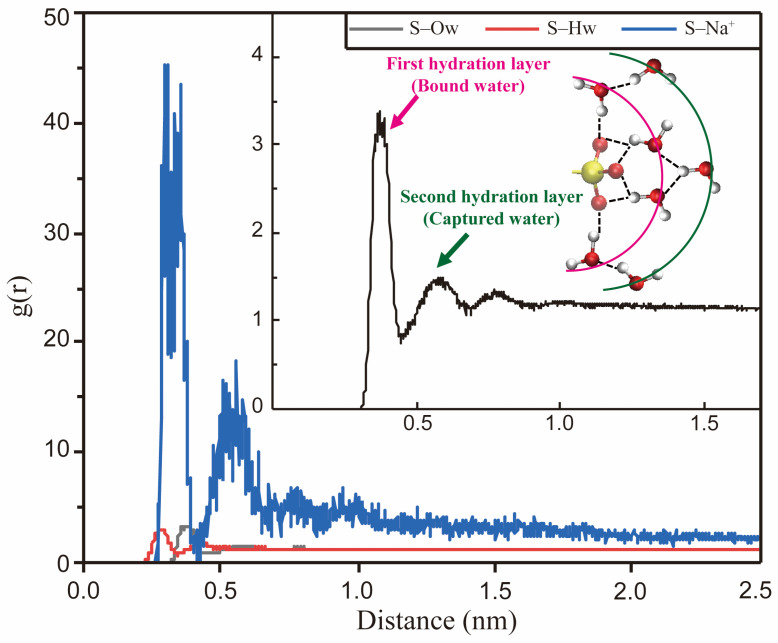
The RDF of the central atom (S) of the IOS surfactant headgroup regarding the hydrogen (Hw) and oxygen atoms (Ow) of the surrounding water molecules and sodium ions (Na^+^) in the water phase. In the inset, the RDF curve is a closeup of the S–Ow curve. The schematic diagram illustrates the hydration structure surrounding the headgroup. The pink arc and arrow indicate the bound water in the hydration shell. The green arc and arrow indicate the captured water in the hydration shell. Oxygen atoms are indicated by red balls, the sulfur atom is indicated by yellow ball, and hydrogen atoms are indicated by silver balls.

The interactions between water molecules and the surfactant headgroups substantially impact the monolayers’ interfacial properties. The ionic surfactant may be immersed several layers deeper into the water phase than the nonionic surfactant. MD simulation method provides detailed information on the hydration shell structure near the monolayers. The spatial distribution function (SDF) can be employed to characterize water molecules’ distribution visually. As shown in Figure 16, compared with dodecyl carboxylate (SDC), SDSn surfactant has more water molecules distributed around the headgroups, indicating the sulfonate group is more hydrophilic than the carboxylate group [86]. Moreover, the RDF can quantitatively characterize the orientation and distribution patterns of the molecules. Figure 17 illustrates the RDF of the central atom (S) on the headgroup of IOS molecules in relation to the oxygen (Ow) and hydrogen (Hw) atoms on the water molecules and sodium ions (Na^+^) in the decane–IOS–water system. The Coulomb force is stronger than the hydrogen bonding effect. The distances between S and Hw atoms are shorter than those of S and Ow atoms (indicated by the horizontal value of the first RDF peak). This means Hw forms hydrogen bonds with oxygen on the headgroups, which determines the orientation of water molecules and forms the hydration shell structure, as illustrated in the inset of Figure 17. Furthermore, the g(r)_S-Ow_ curve has three local peaks representing three hydration layers. The first peak is the sharpest, indicating a strong hydrogen bonding interaction between the headgroup and the water molecules in this range (also known as bound water). The second peak represents the trapping water influenced by the first hydration layer due to the hydrogen bonding effect. The third peak is relatively inconspicuous. The distribution pattern means that the attraction force of surfactant monolayers decreases as the distance increases. The water molecules far away from the interface and unaffected by surfactant monolayers are called free water [39].

**Figure 18:**
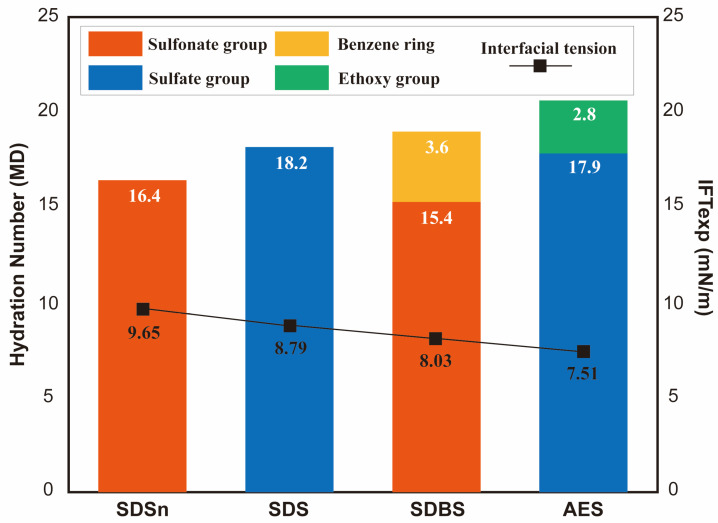
Effects of different hydrophilic headgroups and spacers on the hydration numbers (indicated by bars) and interfacial tensions (indicated by the data points and lines) of the surfactant monolayers. As is observed, a larger hydration number leads to lower IFT values (data are from [76]).

The results of MD simulation studies not only describe the structure of the hydration layers induced by the headgroups but also quantitatively characterize the hydrophilicity of different headgroups by counting the number of solvated water molecules in the vicinity (i.e., coordination number) and by calculating the number of hydrogen bonds formed between the headgroups and the water molecules. Xu et al. [76] investigated the interfacial properties of SDSn, SDS, SDBS, and AES at the dodecane–water interface. The surfactant alkyl tails have the same length. As shown in Figure 18, the hydration number of the sulfate group ($-{SO}_{4}^{-}$) is evidently larger than that of the sulfonate group ($-{SO}_{3}^{-}$). Introducing spacer groups (e.g., benzene rings and ethoxy groups) in the headgroups can further enhance the hydrophilicity. It can also be observed that the IFT at the oil–water interface decreases with the enhancement of the hydrophilicity of surfactants. Zhang et al. [87] used MD simulation to reveal further the influence of spacer groups on the interfacial properties of the surfactants at the air–liquid interfaces. They found that the introduction of some functional groups as spacers into the structure of perfluorooctane sulfonate (PFOS) would not much influence the orientation and conformation of hydrophobic chains in surfactants, while the hydrophilicity of the headgroups would be improved by introducing hydrophilic groups as spacers. As shown in Table 1, compared with PFOS, the average number of hydrogen bonds increases, and the diffusion coefficients of the water molecules in the first shell of the hydrate layer remarkably decrease for PFOS with carbonyl, amino, amide groups, or their combinations. In contrast, the hydrophilicity of the headgroups would not be changed much when the methylene or thioether groups were employed in PFOS as a spacer. 

#### 2.3.2. Influences of Inorganic Salt Ions 

The addition of inorganic salts can reduce the IFT at the oil–water interface [88] and gas–water interface [89] since they can shield the repulsive interactions between the headgroups of the surfactants, therefore enabling the monolayers to become more closely packed. This process can improve the stability of the interface. Liu et al. [90] argued that point charges can represent most inorganic salt ions; therefore, ions with the same charge but different masses have little effect on the interfacial properties of the surfactant monolayers. By contrast, Allen et al. [91] suggested that though different monovalent cations do not change the structure of the monolayers, they can change the interfacial properties to various degrees. They found that the interaction strength of monovalent cations with the headgroups follows the order of ${NH}_{4}^{+}$ > ${Cs}^{+}$ > ${Na}^{+}$ > ${Li}^{+}$. Hu et al. [92] showed that the ability of SDS to reduce IFT increased with the increasing radius of monovalent cations, and the order of IFT reduction follows ${Cs}^{+}$ > ${Rb}^{+}$ > $K^{+}$ > ${Na}^{+}$ > ${Li}^{+}$. Yan et al. [93] showed that divalent ions (${Ca}^{2+}$ and ${Mg}^{2+}$) have a more powerful influence on the hydration structure around the headgroups. They can disturb the original hydrogen bonding structure, leading to a decrease in the hydrogen bond number and an increase in the hydrogen bond lifetime. Compared with ${Ca}^{2+}$, ${Mg}^{2+}$ has much greater difficulty entering the first hydration shell of the headgroups, and once entered into the shell, ${Mg}^{2+}$ has a stronger effect on the hydrogen network. Li et al. [74] reported that the additive ${Ca}^{2+}$ could replace ${Na}^{+}$ at the oil–water interface, which compresses the polarity headgroups of SB12-3 and SDBS so that both surfactants are arranged at the oil–water interface more closely. In the presence of ${Ca}^{2+}$ ions, the interactions between water molecules and sulfonate groups in SB12-3 and SDBS surfactants are enhanced. Meanwhile, ${Na}^{+}$ ions become closer to the sulfonate group in SB12-3, which compresses the thickness of the EDL. Sun et al. [94] pointed out that strong electrostatic interactions between multivalent cations and anionic surfactant molecules are beneficial for the reduction of electrostatic repulsions between the charged headgroups. The cations play a role as a bridge in connecting the surfactant molecules at the surface, improving the accommodation capacity for surfactant molecules, and consequently lowering the IFT and improving the stability of the interface. Zhao et al. [57] give a similar conclusion; meanwhile, they reported that cations would influence the compact degree of SHBS-formed monolayers, and the order follows: ${Ca}^{2+}$ > ${Mg}^{2+}$ > ${Na}^{+}$. 

**Figure 19:**
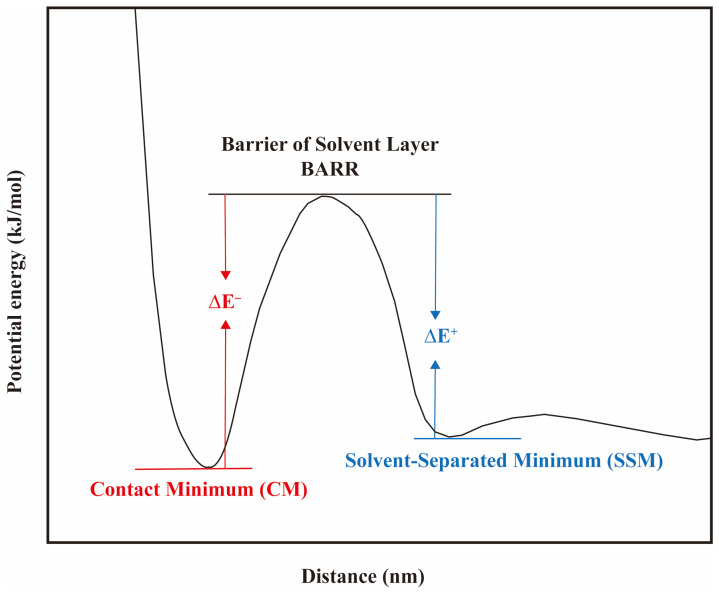
A diagram of the potential of mean force (PMF) between the surfactant headgroups and counterions at the interface. The PMF curve can be predicted using Equation (13).

The combination of hydrophilic headgroups and counterions is defined as ion pairs, which can be bound together by electrostatic attractions. The binding energy of different ion pairs is related to the interaction strength. In MD simulation studies, the energy distribution of the ionic pairs can be determined by calculating the potential of mean force (PMF) [95,96] as shown in the following equation:

(15) $$E\left(r\right)=-k_{B}T\ln g(r)$$

where $k_{B}$ represents the Boltzmann constant, *T* denotes the temperature in the simulations, *g*(*r*) is the RDF of the ionic pairs (refer to Equation (13)). Figure 19 illustrates the PMF curve between ion pairs as a function of the separation distance between them, wherein a peak and two troughs are present. The first trough represents the state of contact minimum (CM), which indicates that the counterions are in direct contact with the headgroup. This state has the lowest energy and the most stable ion pairs. The second trough corresponds to the solvent-separated minimum (SSM), which signifies that the robust hydrogen bonding network formed by the water molecules around the surfactant headgroups hinders the entry of the counterions. Therefore, the counterions located outside the hydration shell are similarly in a relatively stable state. The peak indicates the energy barrier (BARR) of the hydration layers. The counterions must move past the BARR to move into the hydration layers. The BARR is mostly caused by the energy needed to rearrange the water molecules in the layer when the counterions enter the first hydration layer of the surfactants’ hydrophilic headgroups.

With the help of the PMF curves for the ion pairs, we can obtain the binding energy (ΔE^−^ = BARR−CM) as well as the dissociation energy (ΔE^+^ = BARR−SSM) between the counterions and the surfactant headgroups. Based on the ratio *K* of ΔE^−^ to ΔE^+^, the tendency of binding and dissociation of various ion pairs can be discussed. As shown in Table 2, regardless of whether the hydrophilic headgroups are sulfate (${-SO}_{4}^{-}$), sulfonate (${-SO}_{3}^{-}$), or carboxylic acid (${-COO}^{-}$) groups, the energy barriers for ${Na}^{+}$ ions to enter and leave the hydration layers are less than those for ${Ca}^{2+}$ and ${Mg}^{2+}$ ions. That is to say, Na^+^ ions penetrate the hydration layers more easily and come into contact with the hydrophilic headgroups of the surfactants. Meanwhile, it is also easier to dissociate from the layers and return to the aqueous phase in a free state. In contrast, it is difficult for ${Ca}^{2+}$ and ${Mg}^{2+}$ ions to escape from the layers once they enter the hydration shell of the headgroups. Therefore, the interactions between divalent cations and the headgroups are more robust. In summary, on the one hand, the addition of counterions will weaken the structure of water molecules near the hydrophilic headgroups, thus reducing the hydrophilicity of the surfactant. On the other hand, its shielding effect on the electrostatic repulsion between the headgroups will make the interfacial film denser, leading to more surfactant molecules adsorbed at the interface. The increase in the number of surfactant molecules at the interface counterbalances the negative effect of the hydrophilicity weakening of the headgroups [86,93,94,97,98,99].

**Figure 20:**
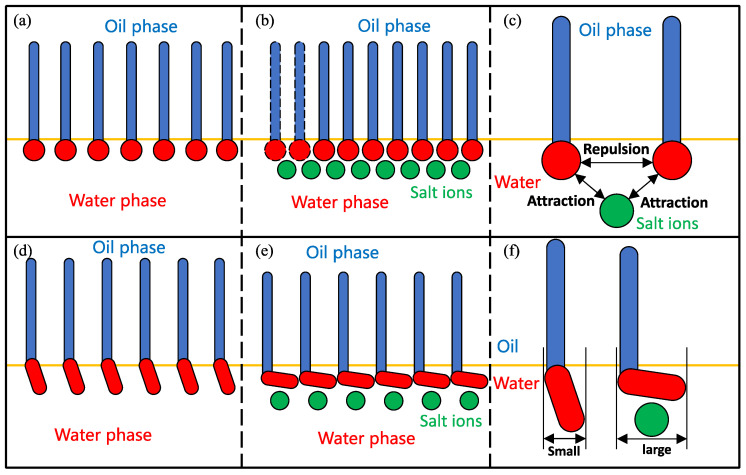
Effect of counterions on the interfacial structures of surfactant monolayers. (**a**) Ionic surfactants at oil–water interface. (**b**) Ionic surfactants and salt ions at oil–water interface. (**c**) The interactions between ionic surfactants and salt ions. (**d**) Nonionic surfactants oil–water interface. (**e**) Nonionic surfactants and salt ions at oil–water interface. (**f**) The interactions between nonionic surfactants and salt ions. The headgroups and tails of the surfactants are represented by red and blue colors, respectively, and salt ions are represented by green balls. The oil–water interface is indicated by the yellow line.

Liu et al. [100,101] reached similar conclusions by measuring the dynamic IFT of surfactant-contained systems. As shown in Figure 20a–c, adding inorganic salt ions increased the number of surfactants transported from the interior of the bulk phase to the interfaces, and increasing the surfactant concentrations at the interface resulted in a reduction in IFT. The decrease in IFT cannot solely be attributed to the increase in interfacial concentration. Alonso et al. [58] found that the addition of inorganic salt ions can also cause a decrease in the IFT of nonionic surfactants while maintaining a constant interfacial concentration.

Compared with ionic surfactants, the headgroups of nonionic surfactants are difficult to completely insert into the aqueous phase due to their relatively weak hydrophilicity and large size; instead, they can only have an inclined orientation at the oil–water interface (see Figure 20d). The addition of inorganic salt ions will generate excessive adsorption of the surfactants at the interface and induce the surfactant headgroups to lie flat at the interface, which will increase the interfacial coverage of the monolayers and further hinder the diffusion and contact between the oil and water molecules. In summary, the presence of inorganic salt ions will not only induce more surfactant molecules to be enriched at the interface but also influence the orientation of the surfactant molecules so that the entire microstructure of the monolayers will be changed, which ultimately enhances the interfacial performance of the surfactants. 

### 2.4. Interactions between Surfactant Alkyl Tails and Hydrophobic Phase

The molecular composition of the hydrocarbon phase is another important factor affecting the interfacial properties of the surfactant-formed monolayers. Chanda et al. [102] found that the monolayer thickness formed at the water–decane interface was 1.25 to 1.3 times higher than that formed at the gas–liquid interface by dodecyl diethylene glycol ether (C12E2). The reason for this is that the strong interactions between decane molecules and the hydrophobic carbon tails of C12E2 led to the straightening of the carbon tails. Under this situation, the surfactant molecules tend to be more perpendicular to the interface, whereas the gas molecules, such as CO_2_ and N_2_, are weakly interacting with the carbon tails, so the surfactant molecules are more randomly oriented at the gas–liquid interface. Moreover, the size of gas molecules is much smaller than that of oil molecules, enabling gas molecules to be more diffused at the interfaces. The contact probability with the water phase is larger than that of oil molecules at the interface, which is detrimental to the IFT reduction. Thus, the molecular configurations of the surfactant monolayers at the gas–liquid interface differs from that at the oil–water interface. Furthermore, Goodarzi and coworkers [103] found that the surfactant tends to stretch more in the case of aliphatic hydrocarbons (octane and dodecane) in comparison to cyclic oil molecules (cyclohexane and benzene) due to the linear structure of the oil molecules. They also found that the IFT is a function of the molecular weight of the hydrocarbons. The difference is attributed to the interaction strength between the hydrocarbon components and the hydrophobic carbon tails of the surfactants [104]. 

The interactions between nonpolar gas molecules (like N_2_, CO_2_, and CH_4_) and the surfactant alkyl tails are controlled by van der Waals forces, which are much weaker than hydrogen bonding and electrostatic interactions. Based on the MD simulation results [16,105], either increasing the interfacial concentration of surfactants or raising pressure can strengthen the interaction forces between the monolayer and the gas phase, leading to increased interfacial width and reduced IFT at the gas–water interfaces. It is in good agreement with experimental work. For example, increasing the concentration of foaming agent SDS and raising the injection pressure of the CO_2_ phase can prolong the half-life period of foam and increase the foaming volume. Sun et al. [106] found that the coalescence and collapse rates of CO_2_ foam are apparently faster than those of N_2_ and O_2_ foam through macroscopic experiments. They simulated the interfacial behaviors of these three foam systems. They found that there were hydrogen bonding interactions between CO_2_/SDS headgroups and water molecules, which weakened the hydrophilicity of SDS and induced self-aggregation of the SDS molecules. This behavior leads to the occurrence of gaps or holes in the monolayers at the interfaces, allowing more water molecules to come into contact with CO_2_ molecules, and eventually leads to a decrease in the stability of the foam liquid film. This mechanism was also demonstrated in foam systems stabilized by dodecyl trimethylammonium bromide (DTAB), nonionic lauryl alkanolamide (LAA) and amphoteric ionic surfactant (CAB), respectively. 

**Figure 21:**
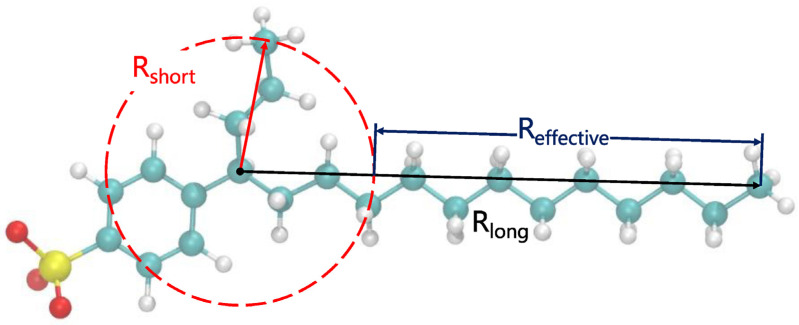
Effective alkyl tail length of the sodium hexadecyl benzene sulfonate (SHBS-4C16). Oxygen atoms are indicated by red balls, the sulfur atom is indicated by a yellow ball, carbon atoms are indicated by cyan balls, and hydrogen atoms are indicated by silver balls (adapted from [52]).

In contrast, it becomes more complicated for oil–water interfaces due to the complex oil components. Wade et al. [68] came up with the idea of the equivalent alkane carbon number (EACN), which is a number that does not have any units and can be used to measure how hydrophobic the oil phase is. It is an important parameter to determine the type and stability of emulsions formed from surfactant–oil–water (SOW) systems. Generally, the EACN is influenced by many factors, such as acyclic, mono- or polycyclic, linear or branched chains, and unsaturated states. It is difficult to estimate from the chemical structure [107,108,109]. Understanding the oil’s EACN value allows us to predict whether this oil should form Winsor type I–III microemulsions under an equilibrium state or a direct/inverse emulsion after stirring [44,108]. At the macroscopic level, the EACN of a particular oil phase is found by looking at how it behaves compared to a well-defined linear hydrocarbon in the same SOW system. Based on the rule that the likes dissolve each other, the IFT of the system would reduce to the minimum when the hydrophobic carbon tails of the surfactants were similar to those of the oil molecules. However, the experimental approaches still face many challenges; for example, they cannot deal with the large number of branched paraffin molecules in the oil displacement system. The problems can be solved using the MD simulation method. Jang et al. [52] proposed effective alkyl tail length (EATL) using the method. As shown in Figure 21, the SHBS surfactant has a twin-tailed structure. The short carbon chain has a shielding effect (i.e., steric hindrance) on the long carbon chain, which prevents the oil molecules from inserting into the space between the two carbon chains. Thus, the oil components mainly interact with the long carbon chains. Under this situation, the EATL of the surfactant alkyl tail (R_effective_) is defined by the difference between the long tail (R_long_) and the short tail (R_short_). In the decane–SHBS–water system, the R_effective_ of SHBS-4C16 is 9.53 ± 1.36 Å, which is very close to the average length of decane molecules (9.97 ± 1.03 Å) in the oil phase. Therefore, SHBS-4C16 has optimum miscibility with the decane phase. The calculations by Xiao and coworkers [110] showed that although SHBS-4C16 has the lowest IFT at the oil–water interface, the EATL is closer to the average length of nonane molecules (see Table 3). In summary, although the EATL method further clarifies the matching relationship between the branched structure in the hydrophobic carbon tails of the surfactants and the oil phase, it still needs to be further studied and improved.

It is well known that crude oil is a complex mixture of n-alkanes, isoparaffins, cycloparaffins, aromatic hydrocarbons, and other nonhydrocarbon constituents [111]. The resin and asphaltene are polar molecules with surface activities [112,113]. Thus, they can adsorb at the interface and affect the interfacial properties. In the past, n-alkanes with a carbon number of 8~14 were always selected as the simulated oil phase in MD simulations, wherein the influence of the strong polar components on the interfacial properties was neglected. A suitable molecular model is essential to mimic the actual complexity of the system. In recent years, researchers have been working hard to continuously upgrade and optimize the molecular models of the crude oil phase to bridge the gap between the simulated results and the experimental work. Kunieda et al. [112] used eight typical types of hydrocarbon molecules, namely hexane, heptane, octane, nonane, cyclohexane, cycloheptane, benzene, and toluene, with different ratios to construct the crude oil model. Sugiyama et al. [114] used quantitative molecular representation (QMR) in combination with experimental approaches to construct a molecular model containing 108 molecules (also termed digital oil), which can successfully characterize the properties and phase behavior of light crude oil produced in domestic Japan. Iwase et al. [115,116] extended the QMR method and successfully constructed a molecular model of heavy oil containing 36 typical types of hydrocarbon molecules. Cui et al. [117] showed bitumen’s microstructural evolution by utilizing a digital oil mode and MD simulations, providing a theoretical framework to elucidate transition states between the liquid and glass states. 

**Figure 22:**
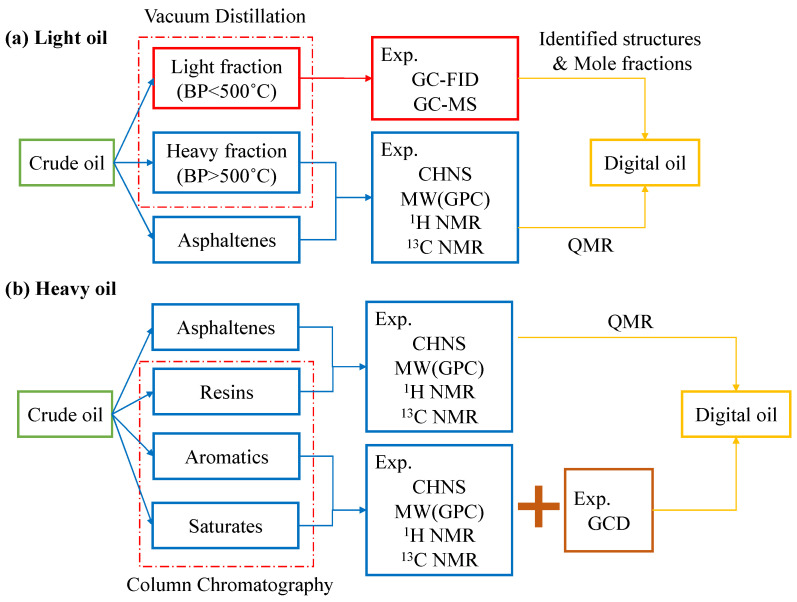
Construction methods for a digital oil model. (**a**) Workflow for light crude oil and (**b**) workflow for heavy crude oil [118].

Figure 22 shows the workflow of constructing digital models of light oil and heavy oil, which can significantly improve the simulated models’ authenticity and help us to further understand the interfacial behaviors of the surfactants at the oil–water interfaces [118]. In addition, several studies have begun to address the imposed effect of polar molecules, such as resin and asphaltene, on their interfacial properties in crude oil. Mizuhara et al. [119] discussed the aggregation behaviors of 13 types of asphaltene molecules at the water–oil (heptane + toluene) interface. They focused on the influence of heteroatoms, such as sulfur and nitrogen, on the adsorption stability of asphaltenes. Gao and coworkers [120,121] investigated the adsorption morphology of the asphaltene molecule C5Pe at the oil–water interface. The authors pointed out that the polycyclic aromatic rings of asphaltene are perpendicular to the interface but not parallel to other asphaltene molecules. The aromatic ring structures on different molecules are aligned parallel to each other by the π–π interaction. The interaction can be weakened by adding inorganic salt ions [35]. However, the carbon chains surrounding the aromatic rings have a steric hindrance effect, which ultimately leads to the formation of irregular agglomerative adsorption of C5Pe at the oil–water interface. At present, there are fewer simulation studies on the effect of strong polar components in crude oil on the IFT and the interfacial structure of the surfactant monolayers. The underlying mechanisms must be further explored and clarified from a microscopic perspective.

## 3. Conclusions and Outlook

The paper gives a comprehensive review of the research progress made in MD simulation studies over the last decade focused on the adsorption behaviors of surfactants at the interface between two immiscible fluids. Initially, the evaluation methods for the interfacial properties and the characterization methods for the microstructures at the interfaces are presented. Balancing the interactions of surfactants with the water and hydrophobic phases can improve the monolayers’ interfacial performance. It can be realized by either enhancing the surfactant–water interactions or surfactant–oil/gas interactions. The methods include increasing the interfacial concentrations (until saturation coverage), introducing chemical groups (such as CO_2_-philic functional group for stabilizing CO_2_ foam), formulating surfactants with twin-tailed structure, and adding cosolvents into surfactants. Consequently, dense and thick monolayers can form, effectively inhibiting the contact between the two immiscible fluids at the interface. The molecular interactions can be classified into three aspects: interactions between the surfactant molecules within the monolayers, interactions between the monolayers and the aqueous phases, and interactions between the monolayers and the hydrophobic phases. The influence factors, such as the molecular structure of the surfactant, the synergistic effect of surfactant mixtures and cosolvents, inorganic salt ions, and the molecular makeup of the hydrocarbon phase, are further analyzed in more detail to see how they affect the morphology and interfacial properties of the monolayers. The main points are listed as follows:

(1) Interactions between the surfactant molecules within the monolayers: with the increase in interfacial concentration, the formed monolayers undergo the process of “GL dispersion–LE phase–LC phase–undulation state–protruding bud structure–restoration of flatness”. In addition, modifying the molecular structure can enhance the interfacial performance of the surfactants. The measures include increasing the size of the headgroups, introducing extra hydrophilic radical groups, polymerizing the monomer molecules, as well as shortening and coarsening the linear-chain molecules. When applying the surfactant mixtures (i.e., synergistic effect), surfactant molecules of small size would be inserted into the gaps between the large surfactant molecules, improving the integrity degree of the monolayers, thus preventing the free diffusion of molecules and the contact between the two immiscible phases.
(2) Interactions between the surfactant monolayers and the water phase: a clear hydration shell (which consists of bound water and captured water) exists near the hydrophilic headgroups of the surfactant. The number of water molecules in the hydrated layers and the number of hydrogen bonds, which quantitatively characterize the hydrophilicity of various headgroups, can be obtained from the MD simulation method. For ionic surfactant molecules, the inorganic salt ions shield the hydrophilic headgroups from electrostatic repulsions, which leads to more surfactant molecules being enriched at the interface. For nonionic surfactant molecules, the salt ions change the orientation of the hydrophilic headgroups, thus improving the degree of interfacial coverage of the monolayers.
(3) Interactions between the surfactant monolayers and the hydrocarbon phase: most of the molecules (such as natural gas, paraffin, and aromatic hydrocarbons) are nonpolar, whereas resins and asphaltene are polar molecules. The nonpolar molecules would interact with the surfactant alkyl tail via van der Waals force. Thus, the molecular configurations at the gas–liquid interface are more disordered. As to nonpolar molecules in the oil phase (such as n-alkanes), the EATL method, using MD simulations, clarifies the matching relationship between the branched structures in the hydrophobic carbon tails and the components of the oil phase. The modeling of the crude oil composition by MD simulations has evolved from the initial pure n-alkanes to multicomponent simulated oils (i.e., digital oil) containing polar compounds. However, the influence of polar molecules with large sizes in crude oil on the interfacial properties of the surfactant monolayers still needs further study.

Based on the above conclusions, the MD simulation method has great potential for studying and analyzing the morphology and properties of various interfaces between two immiscible fluids. To date, most MD simulation studies have employed single-structure surfactants and simplified the oil phase to n-alkanes. With the rapid development of computing power by high-performance clusters and the continuous optimization of modeling approaches, the differences in physicochemical properties between simulated oil phases and natural pore fluids become negligible. To enhance the dependability of MD simulations in predicting the microstructure and thermodynamic characteristics under different conditions and to promote the approach as a widely recognized technology in industrial research and application, it is imperative to improve and innovate it from multiple angles:

(1) Upgrade the spatial and temporal scales. Currently, the dimensions of the simulated systems in most MD simulations are less than 20 nm for the sake of computational efficiency [36]. Expanding the spatial scale of simulations to hundreds of nanometers is crucial to eliminate the randomness of the predictions caused by the size effect. Meanwhile, only if a simulation is ergodic and long enough to allow the system to visit all its energetically relevant states can we derive meaningful information from it [47]. These are beneficial to describe the enrichment process of the surfactant molecules from the interior of the bulk phase to the interface and desorption from the interface under various conditions. Under these circumstances, coarse-grained MD and DPD simulations are recommended [122,123], which can model molecular behaviors from hundreds of nanometers to several micrometers (i.e., with a mesoscopic perspective).
(2) Accurate description of the interface system. Unlike modeling of the bulk phase of the fluids, the intermediate regions in binary fluid systems are heterogeneous. Regarding van der Waals’ interaction, an insufficient cut-off distance for intermolecular interaction would lead to significant artifacts in microstructure and properties at the interfaces [124]. Furthermore, the cut-off scheme’s dispersion correction significantly affects the system’s adsorption process in which the Coulomb force is not strong enough. Lennard-Jones potential with the particle-mesh Ewald (LJ-PME) scheme is a potential solution for this issue [125]. In addition, the commonly used force fields [126,127,128] are developed for specific purposes (e.g., phase behaviors). The simulation results for the interface system may not be quantitatively compared with each other. The existing force fields should be continuously improved with reference to first-principles calculations and experimental values [129,130,131]. The combination of the MD simulation method and machine learning (ML) techniques may provide a fast and cost-effective IFT determination over multiple and complex fluid–fluid and fluid–solid interfaces (i.e., inhomogeneous systems) [132]. The relationship between the IFT, fluid composition, and thermodynamic conditions may involve several variables. In this context, machine learning can be a suitable approach to correlating physical and chemical properties in a single and robust model.

Generally speaking, there is no need to identify and describe every molecule present in reservoir fluids for research and industrial applications. Employing various modeling techniques, from atomistic to mesoscopic scales, to investigate the interfacial behaviors of the surfactants is a highly effective approach [45]. MD simulations based on the first-principles method can simulate the chemical reactions occurring in the reservoir fluids [43]. Coarse-grained MD and DPD simulations can model the surfactant behaviors at the mesoscale [122,123]. Foam flooding experiments and MD simulation studies for the gas–liquid interfaces aim to enhance the foam film’s stability and extend the foam’s half-life period so that they can play a longer and more influential role in oilfield applications. Microemulsion flooding tests and MD simulation studies for the water–oil interface aim to achieve ultra-low IFT to enhance the mobility of the oil phase and the miscibility of the oil and water phases in the reservoirs. The rapid development of simulation technology has complemented the experimental process of the performance evaluation of the surfactants. Suppose there is sufficient experimental data to validate and correlate the computed results. In that case, it is feasible to use molecular modeling computations to forecast the macroscopic behaviors of the systems with a reasonably high level of reliability. In addition, it expedites the advancement of research in improving chemical flooding technologies, which involves the Laplace capillary suction effect, Winsor R theory, hydrophile lipophile balance (HLB) theory, and other related concepts [15,17,44]. Regarding the simulation itself, different theories offer diverse simulation schemes for research. Innovative experimental techniques can provide more precise input parameters and validations for simulated results, therefore advancing the research progress in theoretical simulation methods. To summarize, the development of EOR methods with surfactants needs our continuous efforts and innovations from all the perspectives of theory, experiment, and simulation, as well as further strengthening the links among them. Given the crucial role that interfaces play in porous media for the energy transition, we anticipate this review will also benefit hydrogen storage and energy transitions [133].

## Figures and Tables

**Table 1:** The average number of hydrogen bonds formed between oxygen atoms in the sulfonate group and water molecules in different systems and the diffusion coefficients of water molecules in the first shell of the hydration layer [87].

Surfactants	Average Number of Hydrogen Bonds	Diffusion Coefficients ×10^−6^ (cm^2^/s)
PFOS	1.070	2.588
PFOS-CH_2_	1.081	2.348
PFOS-S	1.107	2.720
PFOS-CO	1.247	1.943
PFOS-NH	1.294	1.942
PFOS-CONH	1.290	1.678

**Table 2:** Predictions of dissociation and binding energy barriers between various ion pairs of counterions and surfactant headgroups (data are from [86,93,94,97]).

Surfactant	Dipolar Pair	CM (kJ/mol)	BARR (kJ/mol)	SSM (kJ/mol)	ΔE^+^ (kJ/mol)	ΔE^−^ (kJ/mol)	K=ΔE^+^/ΔE^−^
SDS	$-{SO}_{4}^{-}-{Na}^{+}$	−5.72	2.70	−2.52	5.22	8.42	0.620
	$-{SO}_{4}^{-}-{Ca}^{2+}$	−7.68	13.38	−3.75	17.13	21.06	0.813
	${-SO}_{4}^{-}-{Mg}^{2+}$	−8.31	28.29	−4.70	32.99	36.60	0.901
SDSn	${-SO}_{3}^{-}-{Na}^{+}$	−5.16	2.56	−2.28	4.84	7.72	0.627
	${-SO}_{3}^{-}-{Ca}^{2+}$	−5.87	18.84	−2.76	21.60	24.71	0.874
	$-{SO}_{3}^{-}-{Mg}^{2+}$	−8.65	29.00	−5.13	34.13	37.65	0.907
SDC	${-COO}^{-}-{Na}^{+}$	-	-	-	6.53	9.60	0.680
	${-COO}^{-}-{Ca}^{2+}$	-	-	-	8.66	17.60	0.492
SDSn	${-SO}_{3}^{-}-{Na}^{+}$	-	-	-	7.78	9.82	0.792
	${-SO}_{3}^{-}-{Ca}^{2+}$	-	-	-	19.12	23.52	0.813
AES + CAB	${-COO}^{-}-{Ca}^{2+}$	−10.32	5.80	−3.00	8.80	16.12	0.546
	${-COO}^{-}-{Mg}^{2+}$	−8.39	8.43	−3.74	12.17	16.82	0.724
AES + DSB	${-SO}_{3}^{-}-{Ca}^{2+}$	−10.59	5.23	−5.45	10.68	15.82	0.675
	$-{SO}_{3}^{-}-{Mg}^{2+}$	−5.74	6.94	−5.58	12.52	12.68	0.987
AES	$-{SO}_{4}^{-}-{Ca}^{2+}$	−31.41	53.31	−19.93	73.24	84.72	0.860
	${-SO}_{4}^{-}-{Mg}^{2+}$	−29.74	85.08	−19.41	104.49	114.82	0.910

Note: CAB is Lauramidopropyl betaine; DSB is dodecyl sulfonate betaine.

**Table 3:** IFT and EATL of SHBS-4C16 surfactant at the n-alkane/water interfaces (data from [52,110]).

Systems	R_nonane_ (nm)	R_decane_ (nm)	R_hendecane_ (nm)	R_short_ (nm)	R_long_ (nm)	R_effective_ (nm)	IFT (mN/m)
nonane + pure water	0.859 ± 0.014			0.370 ± 0.018	1.2241 ± 0.060	0.871 ± 0.078	33.14 ± 0.62
decane + pure water		0.997 ± 0.103		0.384 ± 0.019	1.337 ± 0.135	0.953 ± 0.136	8.02 ± 4.12
hendecane + pure water			1.041 ± 0.023	0.370 ± 0.017	1.231 ± 0.070	0.861 ± 0.087	31.10 ± 3.31
nonane + brine (NaCl)	0.859 ± 0.016			0.372 ± 0.013	1.224 ± 0.086	0.852 ± 0.099	31.41 ± 0.05
decane + brine (NaCl)		0.952 ± 0.018		0.369 ± 0.019	1.240 ± 0.067	0.871 ± 0.086	31.04 ± 0.32
hendecane + brine (NaCl)			1.040 ± 0.022	0.371 ± 0.014	1.229 ± 0.084	0.858 ± 0.098	31.08 ± 3.41

## List of Abbreviations

ABS	Alkyl Benzene Sulfonate
AEC	Alkyl Polyoxyethylene Carboxylate
AES	Sodium Polyoxyethylene Alkyl Ether Sulfate
AOS	Sodium *α*-olefin Sulfonate
BARR	Energy Barrier
CAPB	Cocamidopropyl Betaine
CAB	Lauramidopropyl Betaine
CM	Contact Minimum
CMC	Critical Micelle Concentration
C6C5E3	Triethyleneglyco 6-Dodecyl Ether
C12E2	Dodecyl Diethylene Glycol Ether
C12E3	Triethyleneglycol 1-Dodecyl Ether
DTAB	Dodecyl Trimethylammonium Bromide
DSB	Dodecyl Sulfonate Betaine
EACN	Equivalent Alkane Carbon Number
EATL	Effective Alkyl Tail Length
EDL	Electric Double Layer
EOR	Enhanced Oil Recovery
GPU	Graphics Processing Units
GL	Gas-Like Phase
HLB	Hydrophile Lipophile Balance
HCB	Hydrophilic–CO_2_-philic Balance
IOS	Internal Olefin Sulfonate
IFE	Interface Formation Energy
IFT	Interfacial Tension
LAA	Lauryl Alkanolamide
LAS	Linear Alkylbenzene Sulfonate
LB	Lauryl Betaine
LE	Liquid-Expanded Phase
LC	Liquid-Condensed Phase
LJ	Lennard-Jones Potential
MD	Molecular Dynamics
ML	Machine Learning
NPnAT	Isobaric–Isothermal–Isointerface Area Ensemble
NPDS	Nonylphenol-Substituted Dodecyl sulfonates
ODC	4,4′-Oxydianilinium Chloride
PFOS	Perfluorooctane Sulfonate
PMF	Potent Mean Force
PME	Particle-Mesh Ewald
QMR	Quantitative Molecular Representation
RDF	Radial Distribution Function
Rha-C10-C10	Rhamnolipid
SAPM	Surface Area Per Molecule
SDF	Spatial Distribution Function
SDC	Dodecyl Carboxylate
SDS	Sodium Dodecyl Sulfate
SDSn	Sodium Decyl Sulfonate
SDBS	Sodium Dodecylbenzene Sulfonate
SHBS	Sodium Hexadecane Benzene Sulfonate
SLA	Sodium Laurate
SSM	Solvent-Separated Minimum
SOW	Surfactant–Oil–Water
